# The lack of functional *DNMT2/TRDMT1* gene modulates cancer cell responses during drug-induced senescence

**DOI:** 10.18632/aging.203203

**Published:** 2021-06-17

**Authors:** Dominika Bloniarz, Jagoda Adamczyk-Grochala, Anna Lewinska, Maciej Wnuk

**Affiliations:** 1 Department of Biotechnology, Institute of Biology and Biotechnology, College of Natural Sciences, University of Rzeszow, Rzeszow 35-310, Poland

**Keywords:** DNMT2/TRDMT1, cancer cells, anti-cancer drugs, chemotherapy-induced senescence

## Abstract

Cellular senescence may be a side effect of chemotherapy and other anti-cancer treatments that may promote inflammation and paracrine secondary senescence in healthy tissues. DNMT2/TRDMT1 methyltransferase is implicated in the regulation of cellular lifespan and DNA damage response (DDR). In the present study, the responses to senescence inducing concentrations of doxorubicin and etoposide in different cancer cells with *DNMT2/TRDMT1* gene knockout were evaluated, namely changes in the cell cycle, apoptosis, autophagy, interleukin levels, genetic stability and DDR, and 5-mC and NSUN1-6 levels. Moreover, the effect of azacytidine post-treatment was considered. Diverse responses were revealed that was based on type of cancer cells (breast and cervical cancer, osteosarcoma and glioblastoma cells) and anti-cancer drugs. *DNMT2/TRDMT1* gene knockout in drug-treated glioblastoma cells resulted in decreased number of apoptotic and senescent cells, IL-8 levels and autophagy, and increased number of necrotic cells, DNA damage and affected DDR compared to drug-treated glioblastoma cells with unmodified levels of DNMT2/TRDMT1. We suggest that *DNMT2/TRDMT1* gene knockout in selected experimental settings may potentiate some adverse effects associated with chemotherapy-induced senescence.

## INTRODUCTION

A number of anti-cancer strategies, which are based on chemotherapy, radiotherapy and immunotherapy or the use of CDK4/6 inhibitors and epigenetic modulators may promote cellular senescence in cancer and normal cells and tissues as an adverse side effect [1, 2]. Cellular senescence, a state of permanent cell cycle arrest with well characterized biochemical and molecular biomarkers, is considered to be a tumor suppressor mechanism and tissue repair and regeneration modulator [3, 4]. However, in some circumstances, cellular senescence may also stimulate chronic inflammation and tumorigenesis in aged organisms that is mediated, at least in part, by persistent DNA damage signaling-induced senescence-associated secretory phenotype (SASP) [3–5]. Several signal transduction pathways such as NF-κB, p38 MAPK and C/EBPβ are implicated in the SASP response that result in the secretion of proinflammatory factors such as IL-6, IL-8, CXCL1, CCL2, CCL5 and matrix metalloproteinase (MMP) 3 [6–9]. SASP-mediated proinflammatory milieu may contribute to various aspects of cancer progression, namely may stimulate cancer cell proliferation, migration, invasiveness, angiogenesis and epithelial-mesenchymal transition (EMT) [1, 2]. Moreover, therapy-induced senescence in cancer cells is no longer considered functionally irreversible as senescent cancer cells may escape from growth arrest, resume proliferation and exhibit more invasive and migratory properties than untreated corresponding cancer cells, which in turn may drive the formation of secondary tumors or cancer relapse [1, 2]. Thus, the elimination of cancer-associated senescent cells both cancer cells and normal cells by senolytic drugs and the attenuation of SASP by senostatics seem a new promising approach to prevent or delay cancer recurrence and relieve frailty and multimorbidity in long-term cancer survivors [10, 11].

TRDMT1, tRNA aspartic acid methyltransferase 1, (an alternative previous name DNA methyltransferase 2, DNMT2) is implicated in RNA C-5 methylation that promotes tRNA stability and protein synthesis [12–15]. The biological function of human DNMT2/TRDMT1 is not fully recognized [13], however its role has been proposed during viral infection, RNA processing and stress-mediated modulation of cell proliferation and RNA protection [16–20]. More recently, we have shown that *DNMT2/TRDMT1* gene silencing in human fibroblasts promoted oxidative stress, genomic instability and changes in proliferation-related miRNAs that inhibited cell proliferation and stimulated cellular senescence [21]. There is limited information on DNMT2/TRDMT1-mediated effects on cancer initiation, promotion, progression and therapy [22, 23]. Depletion of *DNMT2/TRDMT1* gene in tumorigenic HEK293 cells resulted in modified mRNA methylation profiles and limited cell proliferation and migration [22]. It was also reported that DNMT2/TRDMT1, a writer of RNA m^5^C at sites of DNA damage, is required for efficient homologous recombination (HR) and provided resistance of U-2 OS osteosarcoma cells to radiotherapy and PARP inhibitors [23]. It was suggested that DNMT2/TRDMT1 might be considered as a novel target in cancer therapy as the loss of DNMT2/TRDMT1 sensitized cancer cells to PARP inhibitors *in vitro* and *in vivo* [23]. It is worthwhile to mention that DNMT2/TRDMT1 activity can be also pharmacologically inhibited as azacytidine (AZA) may diminish DNMT2/TRDMT1-mediated RNA methylation in human cancer cell lines [24]. However, the role of DNMT2/TRDMT1 during chemotherapy-induced senescence and related responses in cancer cells of different origin and type has not been addressed.

In the present study, we have used four different cancer cell types (breast and cervical cancer, osteosarcoma and glioblastoma cells) to analyze the effects of *DNMT2/TRDMT1* gene knockout during doxorubicin- and etoposide-induced senescence and the impact of AZA post-treatment. The loss of *DNMT2/TRDMT1* gene affected drug-induced senescence program in glioblastoma cells that was accompanied by apoptosis resistance, DNA damage and impaired DDR and autophagic response. Detailed consequences of the lack of functional *DNMT2/TRDMT1* gene during drug-mediated senescence in four human cancer cell lines are presented and discussed.

## RESULTS AND DISCUSSION

### *DNMT2/TRDMT1* gene knockout potentiates drug-mediated G2/M cell cycle arrest in breast cancer cells and glioblastoma cells

**Figure 1:**
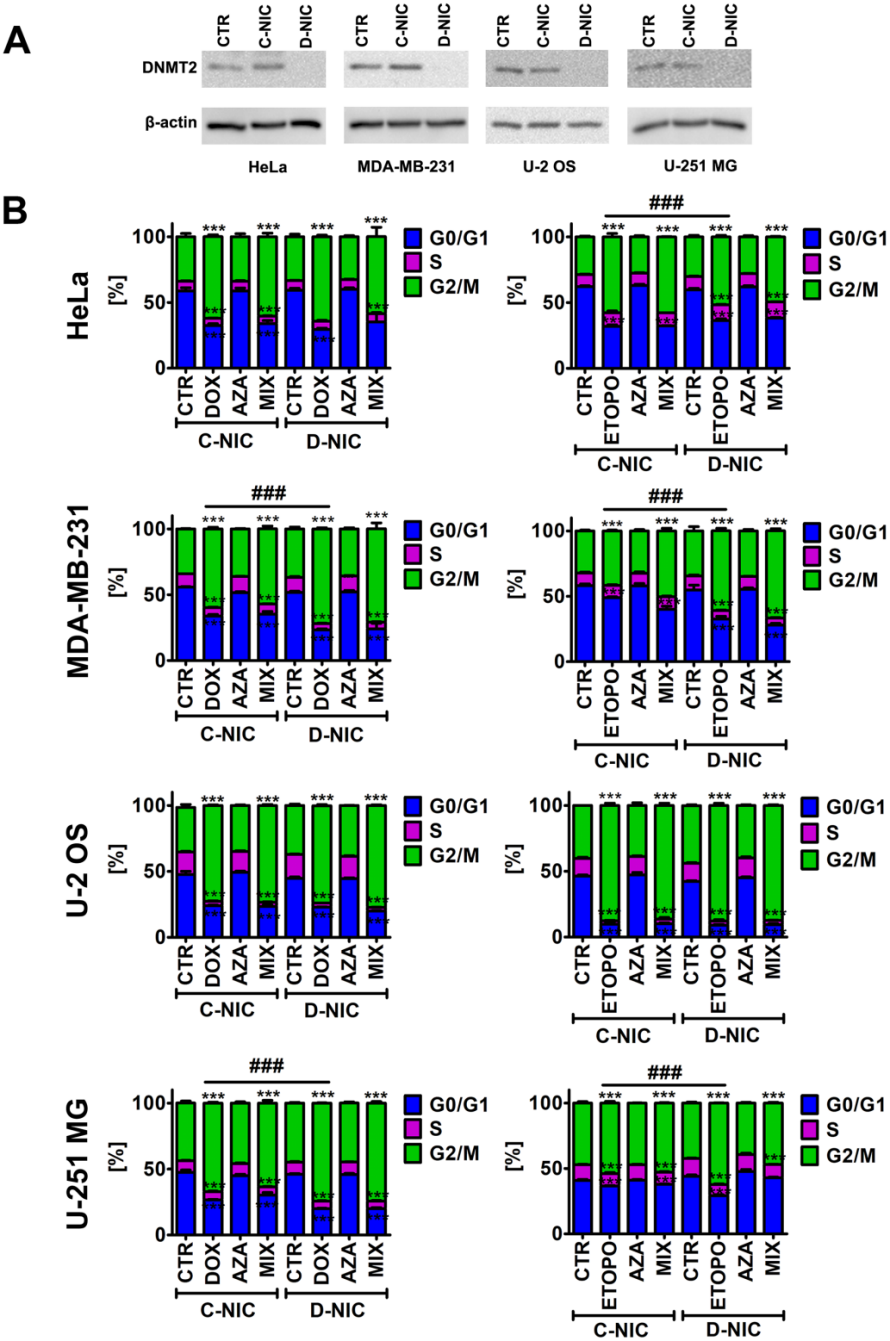
*DNMT2/TRDMT1* gene knockout in four cancer cell lines, namely HeLa cervical cancer cells, MDA-MB-231 breast cancer cells, U-2 OS osteosarcoma cells and U-251 MG glioblastoma cells (**A**) and *DNMT2/TRDMT1* gene knockout-mediated changes in the cell cycle of DOX- and ETOPO-treated cancer cells (**B**). (**A**) Western blot-based analysis of the protein levels of DNMT2/TRDMT1. Anti-β-actin antibody served as a loading control. (**B**) DNA content-based analysis of cell cycle was conducted using flow cytometry. Bars indicate SD, n = 3, ^***^p < 0.001 compared to CTR (ANOVA and Dunnett’s *a posteriori* test), ^###^*p*<0.001 compared to drug-treated C-NIC cells (ANOVA and Tukey’s *a posteriori* test). CTR, control conditions; DOX, doxorubicin treatment; ETOPO, etoposide treatment; AZA, azacytidine treatment; MIX, azacytidine post-treatment; C-NIC, control cells with unmodified levels of DNMT2/TRDMT1 containing control plasmid; D-NIC, cells with *DNMT2/TRDMT1* gene knockout containing dedicated DNMT2 double nickase plasmid.

To address a universal role of DNMT2/TRDMT1 methyltransferase during anti-cancer drug-induced senescence, four different cancer cell lines were used, namely MDA-MB-231 breast cancer cells, HeLa cervical cancer cells, U-2 OS osteosarcoma cells and U-251 MG glioblastoma cells, and CRISPR/Cas9 technology was considered to knockout the *DNMT2/TRDMT1* gene. Two different DNMT2 double nickase plasmids (two target-specific 20 nt guide RNAs, gRNAs) were used and two different clones with non-detectable levels of DNMT2/TRDMT1 were obtained. A representative Western blot-based analysis of DNMT2 levels is presented in Figure 1A.

Similar *DNMT2/TRDMT1* gene knockout efficiency using two different target-specific gRNAs was obtained (data not shown) and further experiments were conducted using two different clones. As similar results were noticed, representative data on selected clone are presented (D-NIC cells). As control cells, cells transfected with control double nickase plasmid (non-targeting 20 nt scramble guide RNA, C-NIC cells) were used.

To promote chemotherapy-induced senescence, two anti-cancer drugs were considered, namely topoisomerase II inhibitors 35 nM doxorubicin (DOX) and 1 μM etoposide (ETOPO) and 24 h treatment. The concentrations were selected on the basis of literature data [1, 25]. Fibrosarcoma, colon cancer and ovarian cancer cells were treated with DOX at the concentrations ranging from 20 to 50 nM and increased senescence-associated beta-galactosidase (SA-β-gal) activity, G1 cell cycle arrest and elevated micronuclei formation were observed [1, 25]. Similar effects were noticed when fibrosarcoma cells were stimulated with 900 nM ETOPO [1, 25]. In our experimental conditions, treatment with both anti-cancer drugs resulted in G2/M cell cycle arrest of the cell cycle regardless of cancer cell type (*p* < 0.001, Figure 1B). *DNMT2/TRDMT1* gene knockout potentiated this effect in MDA-MB-231 breast cancer cells and U-251 MG glioblastoma cells (*p* < 0.001, Figure 1B). In contrast, a minor increase in G0/G1 cell subpopulation was noticed in ETOPO-treated HeLa cells with *DNMT2/TRDMT1* gene knockout compared to ETOPO-treated control HeLa cells (*p* < 0.001, Figure 1B). The effect of 1 μM 5-azacytidine (AZA, a hypomethylating agent with anti-cancer activity [26]) alone and in combination with anti-cancer drugs (post-treatment) was also studied (Figure 1B and Supplementary Figure 1). However, AZA alone did not affect the cell cycle of cancer cells and did not potentiate the effect of anti-cancer drugs in D-NIC cells (Figure 1B and Supplementary Figure 1). AZA and its deoxy derivative 5-aza-2’-deoxycytidine (decitabine) can be considered both effective in inhibiting DNA methylation, and 1 μM azacytidine, but not 1 μM decitabine, can also inhibit cytosine 38 methylation of tRNA^Asp^, a major substrate of DNMT2/TRDMT1, in different human cancer cell lines [24]. Thus, we have used relatively low sub-toxic concentration of 1 μM azacytidine to analyze the effects of AZA-mediated changes in RNA methylation on C-NIC and D-NIC cell proliferation and viability as a single and combined treatment with DOX or ETOPO (AZA post-treatment). Of course, AZA can also promote cytotoxic effects in human cancer cells, for example, apoptosis induction, when used at higher concentrations ranging from 5 to 50 μM [27].

### *DNMT2/TRDMT1* gene knockout limits drug-induced p21-mediated senescence in glioblastoma cells

**Figure 2:**
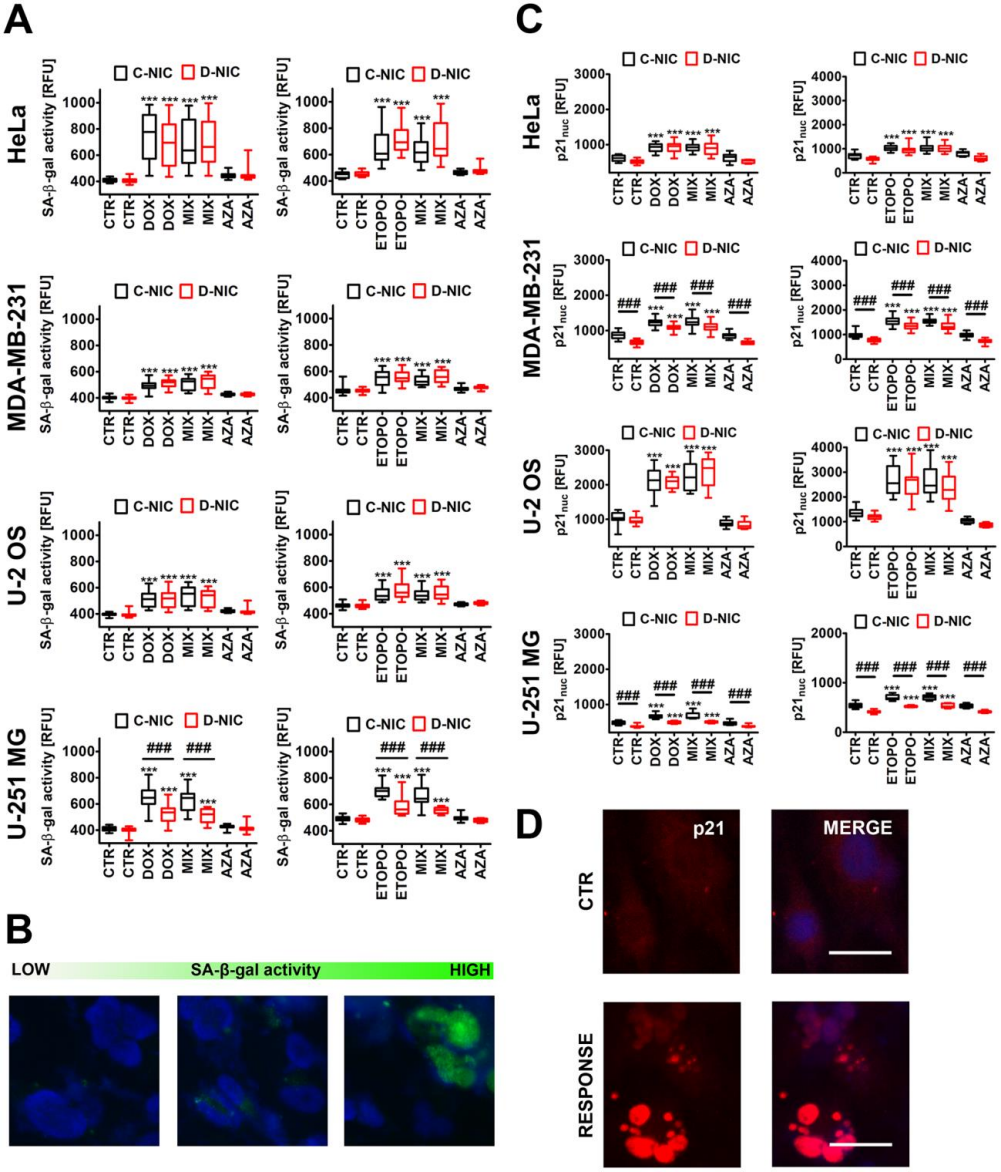
*DNMT2/TRDMT1* gene knockout-mediated changes in the levels of senescence-associated-beta-galactosidase (SA-β-gal) positive cells (**A**, **B**) and nuclear p21 pools (**C**, **D**) in four cancer cell lines treated with DOX or ETOPO. (**A**, **B**) Cellular senescence was revealed using imaging cytometry. SA-β-gal activity is presented as relative fluorescence units (RFU). (**B**) Representative microphotographs are shown, objective 20x, nucleus staining (blue), SA-β-gal staining (green). (**C**, **D**) Nuclear p21 immuno-staining. (**C**) Nuclear p21 levels are presented as relative fluorescence units (RFU). (**D**) Representative microphotographs are shown, objective 20x, nucleus staining (blue), nuclear p21 immuno-staining (red), RESPONSE, representative DOX or ETOPO treatment. (**A**, **C**) Box and whisker plots are shown, n = 3, ^***^p < 0.001 compared to CTR (ANOVA and Dunnett’s *a posteriori* test), ^###^*p*<0.001 compared to C-NIC cells at the same culture conditions (ANOVA and Tukey’s *a posteriori* test). CTR, control conditions; DOX, doxorubicin treatment; ETOPO, etoposide treatment; AZA, azacytidine treatment; MIX, azacytidine post-treatment; C-NIC, control cells with unmodified levels of DNMT2/TRDMT1 containing control plasmid; D-NIC, cells with *DNMT2/TRDMT1* gene knockout containing dedicated DNMT2 double nickase plasmid.

We have then asked the question of whether observed cell cycle arrest might be considered permanent. Thus, we have analyzed drug-induced cellular senescence and the effect of *DNMT2/TRDMT1* gene knockout (Figure 2).

DOX and ETOPO promoted senescence-associated beta-galactosidase (SA-β-gal) activity and an increase in nuclear p21 levels in four cancer cell lines both in C-NIC and D-NIC cells (*p* < 0.001, Figure 2A, 2C). *DNMT2/TRDMT1* gene knockout modulated senescence biomarkers only in glioblastoma cells (*p* < 0.001, Figure 2A, 2C). There was a decrease of 30% and 20% of SA-β-gal positive D-NIC glioblastoma cells compared to C-NIC glioblastoma cells upon DOX and ETOPO treatments, respectively (*p* < 0.001, Figure 2A). *DNMT2/TRDMT1* gene knockout also promoted a decrease in nuclear p21 pools in DOX- and ETOPO-treated glioblastoma D-NIC cells compared to DOX- and ETOPO-treated glioblastoma C-NIC cells (*p* < 0.001, Figure 2C). 1 μM AZA alone did not induce cellular senescence and did not potentiate the pro-senescent effect of DOX and ETOPO in four cancer cell lines both in C-NIC and D-NIC cells (Figure 2). It was previously reported that 20 μM decitabine, but not 20 μM azacytidine promoted a senescence-like phenotype in *in vitro* and *in vivo* cancer models when used for 72 h [27]. The authors concluded that two closely related DNMT inhibitor (DNMTi) nucleoside analogues azacytidine and decitabine may induce a substantially different molecular responses in cancer cells, namely azacytidine may trigger apoptosis, whereas decitabine may stimulate cellular senescence [27]. Indeed, decitabine treatment was associated with the induction of senescence program in diverse cancer cells as judged by increased SA-β-gal activity, reduced proliferation, p16/p21/p27 upregulation, SASP induction or DNA damage response [1, 28–30].

### *DNMT2/TRDMT1* gene knockout decreases drug sensitivity in glioblastoma cells

**Figure 3:**
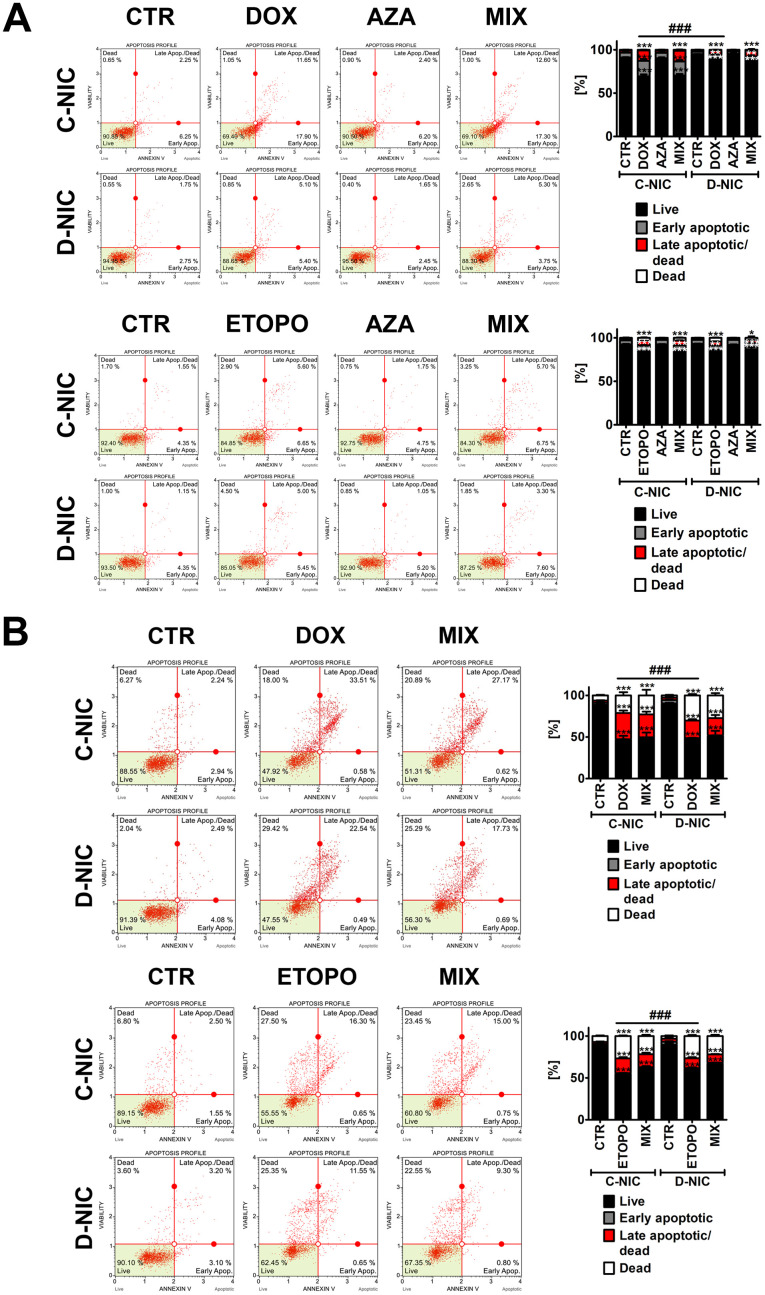
*DNMT2/TRDMT1* gene knockout-mediated apoptosis and necrosis in U-251 MG glioblastoma cells treated with DOX or ETOPO for 24 h and AZA post-treatment for 24 h and immediately assayed (**A**) and assayed after 7 days of drug removal and AZA post-treatment for 24 h (**B**). Apoptosis and necrosis were analyzed using flow cytometry. Representative dot plots are shown. Bars indicate SD, n = 3, ^***^p < 0.001, ^**^p < 0.01, ^*^p < 0.05 compared to CTR (ANOVA and Dunnett’s *a posteriori* test), ^###^*p* < 0.001 compared to drug-treated C-NIC cells (ANOVA and Tukey’s *a posteriori* test). CTR, control conditions; DOX, doxorubicin treatment; ETOPO, etoposide treatment; AZA, azacytidine treatment; MIX, azacytidine post-treatment; C-NIC, control cells with unmodified levels of DNMT2/TRDMT1 containing control plasmid; D-NIC, cells with *DNMT2/TRDMT1* gene knockout containing dedicated DNMT2 double nickase plasmid.

Despite the fact that we have used relatively low concentrations of anti-cancer drugs promoting senescence in cancer cells [1, 25], we have then decided to evaluate DOX- and ETOPO-induced apoptosis (Annexin V staining) and the effect of *DNMT2/TRDMT1* gene knockout after 24 h of drug treatments and 7 days after drug removal (Figure 3 and Supplementary Figures 2, 3).

U-2 OS and U-251 MG D-NIC cells were more resistant to 24 h treatment with DOX compared to corresponding DOX-treated C-NIC cells (*p* < 0.001, Figure 3A and Supplementary Figure 2A), whereas HeLa and MDA-MB-231 D-NIC cells were more sensitive to 24 h treatment with DOX compared to corresponding DOX-treated C-NIC cells (*p* < 0.001, Supplementary Figure 2A). It was previously reported that a primary response to 1 μM DOX treatment for 2 h is apoptotic cell death in breast cancer cells [31]. However, the residual surviving population is subjected to accelerated senescence program that is manifested by the upregulation of p21, downregulation of cdc2 and the involvement of reactive oxygen species (ROS) regardless of p53 status [31]. The changes in phosphatidylserine externalization between ETOPO-treated C-NIC and D-NIC cells were rather mild and insignificant (Figure 3A and Supplementary Figure 2B). One exception was ETOPO treatment in U-2 OS D-NIC cells that resulted in moderate increase in ETOPO sensitivity compared to corresponding ETOPO-treated U-2 OS C-NIC cells (*p* < 0.001, Supplementary Figure 2B). AZA did not promote apoptosis and did not affect DOX- and ETOPO-induced apoptotic cell death in four cancer cell lines (Figure 3A and Supplementary Figure 2). HeLa and MDA-MB-231 D-NIC cells were also more prone to apoptotic cell death after 7 days of drug removal compared to corresponding DOX-treated C-NIC cells (Supplementary Figure 3A). In contrast, U-2 OS and U-251 MG D-NIC cells were less susceptible to apoptosis after 7 days of drug removal compared to corresponding DOX-treated C-NIC cells (Figure 3B and Supplementary Figure 3A). Moreover, U-2 OS and U-251 MG D-NIC cells were more sensitive to necrosis after 7 days of ETOPO and DOX removal, respectively (*p* < 0.001, Figure 3B and Supplementary Figure 3). There were 12% and 10% more necrotic U-2 OS and U-251 MG D-NIC cells compared to corresponding drug-treated C-NIC cells after 7 days of ETOPO and DOX removal, respectively (*p* < 0.001, Figure 3B and Supplementary Figure 3). We have also analyzed senolytic activity of AZA after 7 days of drug removal and treated senescent cancer cells with AZA for 24 h. Surprisingly, *DNMT2/TRDMT1* gene knockout resulted in AZA-mediated apoptosis-based senolytic activity in HeLa D-NIC cells compared to corresponding C-NIC cells upon stimulation with DOX and ETOPO (Supplementary Figure 3). In contrast, AZA-mediated senolytic activity relied on necrosis in ETOPO-treated U-2 OS D-NIC cells (Supplementary Figure 3). Thus, in these particular cellular settings, *DNMT2/TRDMT1* gene knockout may be considered as a therapeutic strategy to eliminate senescent cancer cells. However, AZA-mediated senolytic effects were not observed in drug-treated MDA-MB-231 and U-251 MG D-NIC cells compared to corresponding C-NIC cells (Figure 3B and Supplementary Figure 3).

### *DNMT2/TRDMT1* gene knockout augments drug-induced oxidative stress in glioblastoma cells

As DOX and ETOPO anti-cancer action may be mediated or accompanied by oxidative stress [31–35], the levels of superoxide were also examined after drug treatment and *DNMT2/TRDMT1* gene knockout (Supplementary Figure 4). Indeed, DOX and ETOPO stimulations resulted in increased levels of superoxide in four cancer cell lines (Supplementary Figure 4). DOX-induced oxidative stress may stimulate apoptosis in different cancer cells [33, 34], but low-dose DOX treatment (a subclinical concentration of 100 nM) may also promote oxidative stress-mediated invasiveness in U-2 OS osteosarcoma cells [36]. The free radical scavengers, glutathione (GSH) and *N*-acetyl cysteine (NAC) may also attenuate DOX-induced senescence response in MCF-7 and MDA-MB-231 breast cancer cells [31]. ETOPO promoted a selective non-apoptotic cell death in oncogenic Akt-transduced U-87 MG glioblastoma cells that was mediated by elevated ROS production and oxidative damage [35]. *DNMT2/TRDMT1* gene knockout also potentiated drug-induced oxidative stress in glioblastoma cells, whereas in DOX- and ETOPO-treated U-2 OS D-NIC cells, the levels of superoxide were decreased compared to corresponding drug-treated C-NIC cells (*p* < 0.001, Supplementary Figure 4). AZA did not induce oxidative stress and did not augment drug-induced superoxide production in four cancer cell lines (Supplementary Figure 4).

### *DNMT2/TRDMT1* gene knockout aggravates DOX-induced DSBs and impairs DDR in glioblastoma cells

**Figure 4:**
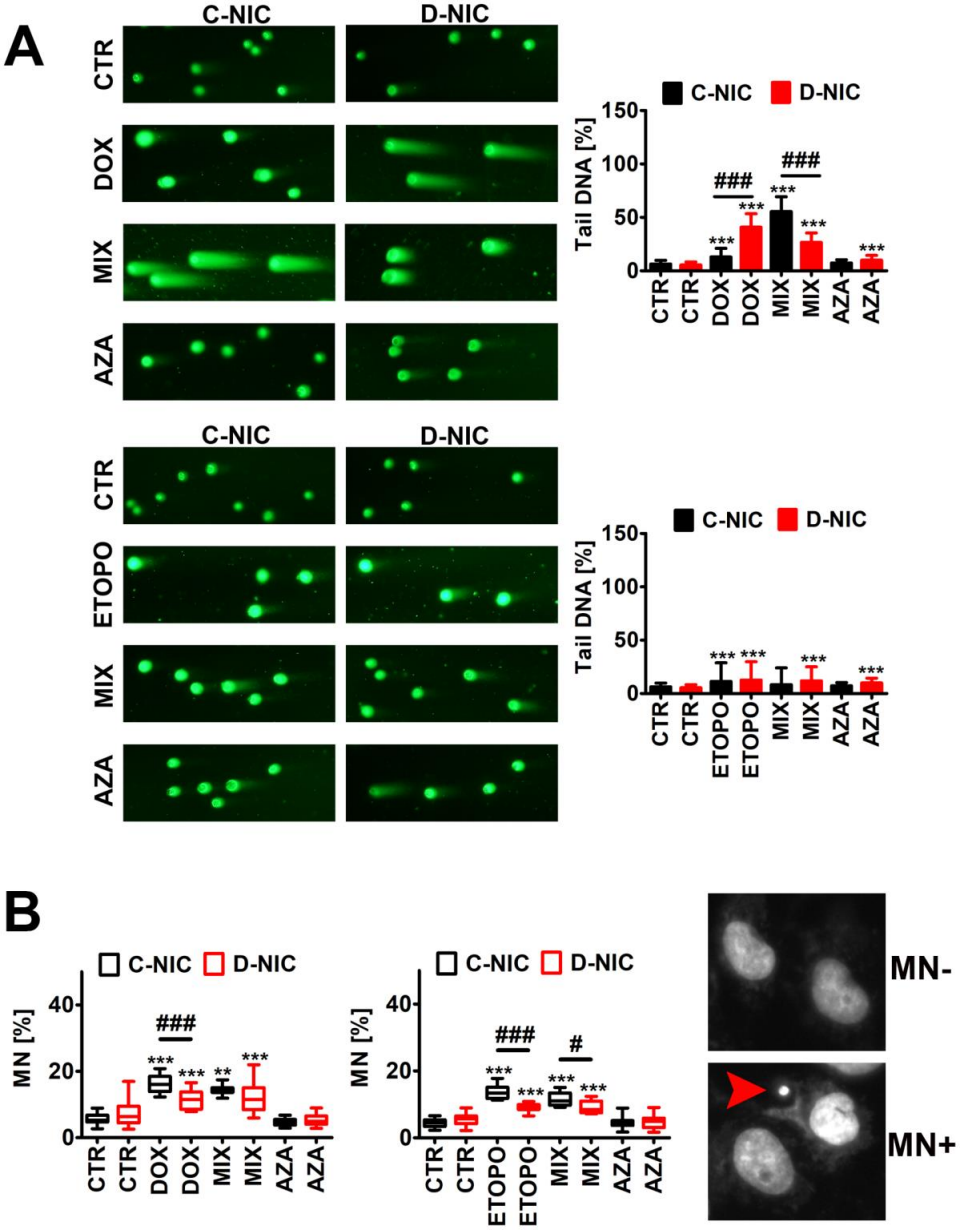
*DNMT2/TRDMT1* gene knockout-mediated DNA damage (**A**) and chromosomal damage (**B**) in U-251 MG glioblastoma cells treated with DOX or ETOPO. (**A**) DNA double-strands breaks (DSBs) as tail DNA (%) were assessed using neutral comet assay. Representative microphotographs are shown, objective 10x, DNA staining (green). Bars indicate SD, n = 3, ^***^p < 0.001 compared to CTR (ANOVA and Dunnett’s *a posteriori* test), ^###^*p* < 0.001 compared to C-NIC cells at the same culture conditions (ANOVA and Tukey’s *a posteriori* test). (**B**) Micronuclei (MN) formation was assayed using Hoechst 33342 staining and scored as % (a red arrowhead). Box and whisker plots are shown, n = 3, ^***^p < 0.001, ^**^p < 0.01 compared to CTR (ANOVA and Dunnett’s *a posteriori* test), ^###^*p* < 0.001, ^#^*p* < 0.05 compared to C-NIC cells at the same culture conditions (ANOVA and Tukey’s *a posteriori* test). CTR, control conditions; DOX, doxorubicin treatment; ETOPO, etoposide treatment; AZA, azacytidine treatment; MIX, azacytidine post-treatment; C-NIC, control cells with unmodified levels of DNMT2/TRDMT1 containing control plasmid; D-NIC, cells with *DNMT2/TRDMT1* gene knockout containing dedicated DNMT2 double nickase plasmid.

**Figure 5:**
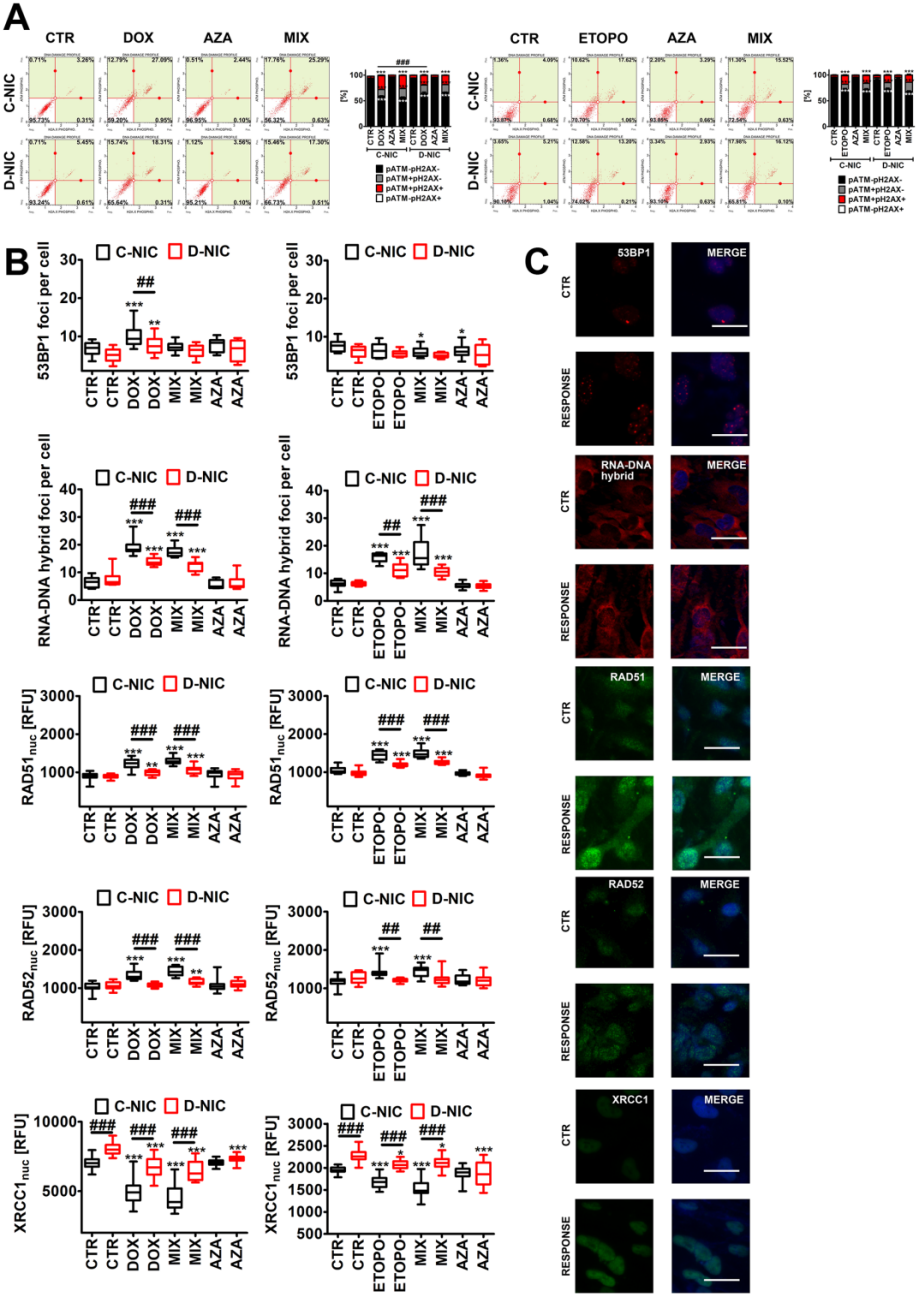
***DNMT2/TRDMT1* gene knockout-mediated DNA damage response (DDR) in U-251 MG glioblastoma cells treated with DOX or ETOPO.** (**A**) Activation of ATM and H2AX was evaluated using flow cytometry. Representative dot plots are shown. Bars indicate SD, n = 3, ^***^p < 0.001 compared to CTR (ANOVA and Dunnett’s *a posteriori* test), ^###^*p* < 0.001 compared to drug-treated C-NIC cells (ANOVA and Tukey’s *a posteriori* test). (**B**, **C**) 53BP1 foci, RNA-DNA hybrid foci, RAD51, RAD52 and XRCC1 immunostaining (red or green). The levels of RAD51, RAD52 and XRCC1 are expressed as relative fluorescence units (RFU). Representative microphotographs are shown, objective 20x, nucleus staining (blue), RESPONSE, representative DOX or ETOPO treatment. Box and whisker plots are shown, n = 3, ^***^p < 0.001, ^**^p < 0.01, ^*^p < 0.05 compared to CTR (ANOVA and Dunnett’s *a posteriori* test), ^###^*p* < 0.001, ^##^*p* < 0.01 compared to C-NIC cells at the same culture conditions (ANOVA and Tukey’s *a posteriori* test). CTR, control conditions; DOX, doxorubicin treatment; ETOPO, etoposide treatment; AZA, azacytidine treatment; MIX, azacytidine post-treatment; C-NIC, control cells with unmodified levels of DNMT2/TRDMT1 containing control plasmid; D-NIC, cells with *DNMT2/TRDMT1* gene knockout containing dedicated DNMT2 double nickase plasmid.

As TRDMT1/DNMT2 was recently proposed to be involved in DNA damage response (DDR) in cancer cells [23], we have then analyzed the effect of *DNMT2/TRDMT1* gene knockout on genetic instability (Figure 4 and Supplementary Figure 5) and DDR (Figure 5 and Supplementary Figure 5) during drug-induced senescence in four cancer cell lines.

Except of DOX-treated U-2 OS cells, DOX and ETOPO promoted DNA double strand breaks (DSBs) in four cancer cell lines (Figure 4A and Supplementary Figure 5). U-251 MG D-NIC cells were the most prone to DNA damage upon DOX stimulation (Figure 4A and Supplementary Figure 5A). There was a 3.5-fold increase in the levels of DSBs in DOX-treated U-251 MG D-NIC cells compared to DOX-treated U-251 MG C-NIC cells (*p* < 0.001, Figure 4A). In contrast, in ETOPO-treated U-2 OS D-NIC cells, a 2-fold decrease in the levels of DSBs was revealed compared to ETOPO-treated U-2 OS C-NIC cells (*p* < 0.001, Supplementary Figure 5C). These effects were associated with different DDR in U-251 MG and U-2 OS D-NIC cells (Figure 5 and Supplementary Figure 5). Drug-treated U-2 OS D-NIC cells were characterized by increased levels of cells with dual activation of ATM and H2AX (elevated phosphorylated forms of ATM and H2AX) compared to corresponding drug-treated C-NIC cells (*p* < 0.001, Supplementary Figure 5B, 5D). In contrast, impaired DDR was revealed in drug-treated U-251 MG D-NIC cells that was judged by decreased phosphorylation status of ATM and H2AX, limited formation of 53BP1 foci and RNA-DNA hybrid foci, and reduced levels of RAD51 and RAD52 compared to corresponding drug-treated C-NIC cells (*p* < 0.001, Figure 5). More recently, RNA modification-orchestrated TRDMT1-m^5^C-RAD52-RAD51 axis promoting homologous recombination (HR) at ROS-induced DSBs was described [23]. DNMT2/TRDMT1 was shown to be recruited to DNA damage sites and required for the induction of RNA m^5^C as a writer of RNA m^5^C at sites of DNA damage [23]. Loss of DNMT2/TRDMT1 affected HR, a pathway critical for DSB repair, by impaired localization of RAD51 and RAD52 to ROS-induced DNA damage [23]. RAD52 was also established to be a reader of RNA m^5^C as RAD52 showed a higher affinity for RNA-DNA hybrids containing m^5^C modified RNA than hybrids without the modification [23]. Moreover, the lack of functional *DNMT2*/*TRDMT1* gene potentiated cancer cell sensitivity to radiation and PARP inhibitors (PARPi) [23]. Except of AZA-mediated synergistic effect on DOX-induced DNA damage in U-251 MG C-NIC cells, AZA post-treatment did not significantly augment drug-stimulated DNA damage (Figure 4A and Supplementary Figure 5). It was proposed that azacytidine has limited ability to induce DSBs and micronuclei formation compared to decitabine in different cancer models *in vitro* [27]. DOX and ETOPO also promoted micronuclei production, but *DNMT2/TRDMT1* gene knockout did not potentiate this effect in four cancer cell lines (Figure 4B and Supplementary Figure 5A, 5C). Moreover, XRCC1 did not seem to play an important role during DOX- and ETOPO-induced DDR in four cancer cell lines as judged by rather mild and insignificant changes in its levels. In general, XRCC1 levels decreased in our experimental settings (Figure 5B and Supplementary Figure 5A, 5C). In DOX-treated U-251 MG cells, the levels of 53BP1 and XRCC1 were negatively correlated (Figure 5B). DOX-treated U-251 MG D-NIC cells were characterized by decreased pools of 53BP1 and increased pools of XRCC1 compared to corresponding DOX-treated C-NIC cells (*p* < 0.001, Figure 5B). Indeed, 53BP1 is considered a negative regulator of alternative non-homologous end joining (NHEJ) pathway that involves XRCC1 protein [37]. As DNMT2/TRDMT1- or NSUN2-mediated methylation may modulate the interactions between tRNAs and Argonautes (Agos) to produce tRNA-related fragments (tRFs) controlling gene expression by transcriptional, post-transcriptional and translational regulation during stress conditions [38–41], we have also analyzed the levels of Ago2 during drug-induced senescence and the effect of *DNMT2/TRDMT1* gene knockout (Supplementary Figure 6). DOX and ETOPO stimulations resulted in an increase in the levels of Ago2 in HeLa and MDA-MB-231 cells, but there were no differences between C-NIC and D-NIC cells (*p* < 0.001, Supplementary Figure 6). Thus, one can conclude that *DNMT2/TRDMT1* gene knockout did not affect Ago2-mediated regulatory role during stress-promoted changes in gene expression profiles.

### *DNMT2/TRDMT1* gene knockout diminishes drug-induced autophagy in glioblastoma cells

**Figure 6:**
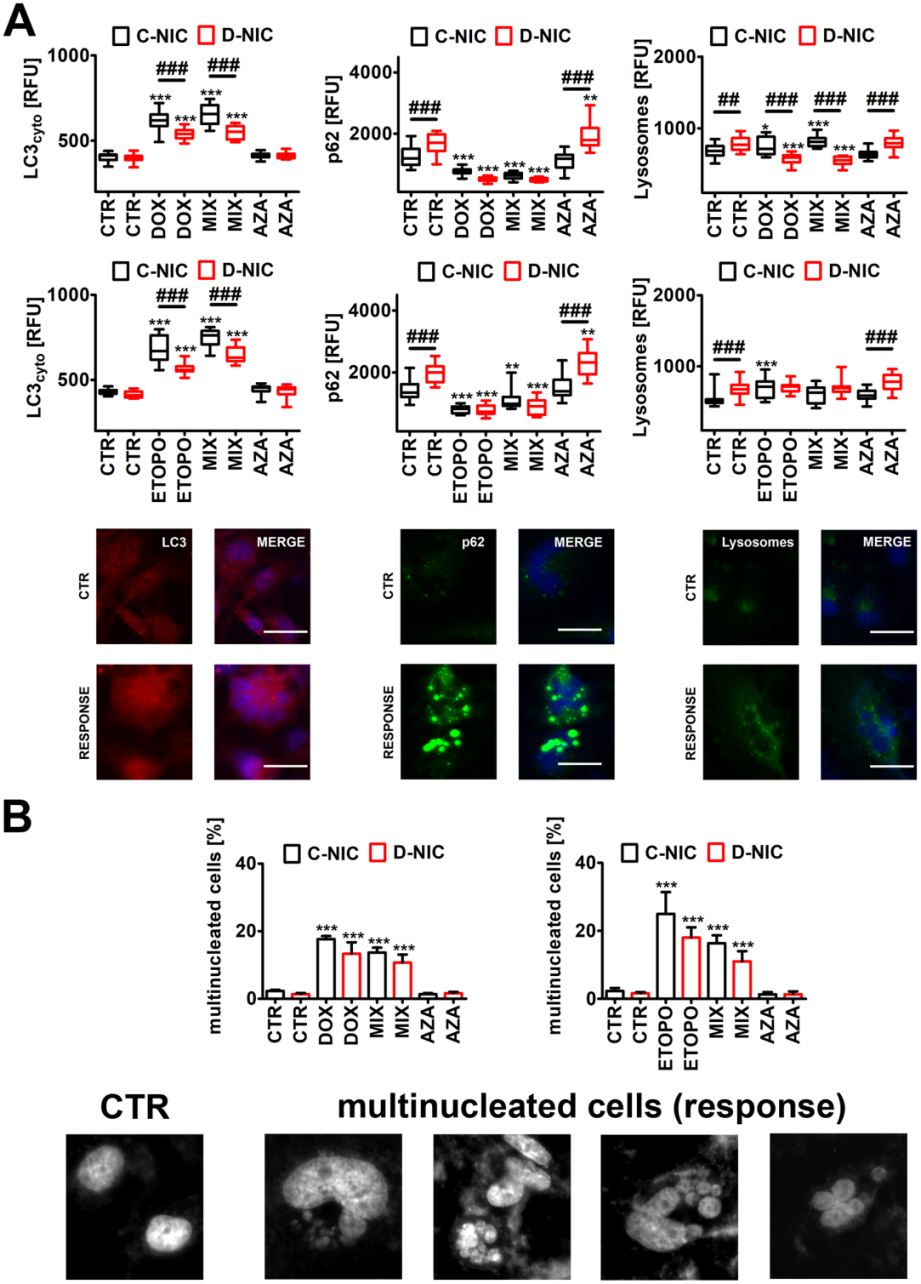
*DNMT2/TRDMT1* gene knockout-mediated autophagy (**A**) and multinucleation (**B**) in U-251 MG glioblastoma cells treated with DOX or ETOPO. (**A**) LC3 immunostaining (red) and GFP-based imaging of a lysosomal marker Lamp1 and an autophagy marker p62 (green). The levels of LC3, p62 and Lamp1 are expressed as relative fluorescence units (RFU). Representative microphotographs are shown, objective 20x, nucleus staining (blue), RESPONSE, representative DOX or ETOPO treatment. Box and whisker plots are shown, n = 3, ^***^p < 0.001, ^**^p < 0.01, ^*^p < 0.05 compared to CTR (ANOVA and Dunnett’s *a posteriori* test), ^###^*p* < 0.001, ^##^*p* < 0.01 compared to C-NIC cells at the same culture conditions (ANOVA and Tukey’s *a posteriori* test). (**B**) Multinucleation events (%) were analyzed using Hoechst 33342 staining. Representative microphotographs are shown, objective 20x, RESPONSE, representative DOX or ETOPO treatment. Bars indicate SD, n = 3, ^***^p < 0.001 compared to CTR (ANOVA and Dunnett's *a posteriori* test). CTR, control conditions; DOX, doxorubicin treatment; ETOPO, etoposide treatment; AZA, azacytidine treatment; MIX, azacytidine post-treatment; C-NIC, control cells with unmodified levels of DNMT2/TRDMT1 containing control plasmid; D-NIC, cells with *DNMT2/TRDMT1* gene knockout containing dedicated DNMT2 double nickase plasmid.

DOX and ETOPO promoted autophagy in four cancer cell lines as judged by increased levels of cytosolic LC3 (*p* < 0.001, Figure 6A and Supplementary Figure 7).

However, in DOX- and ETOPO-treated U-251 MG D-NIC cells, the levels of cytosolic LC3 were significantly decreased compared to corresponding drug-treated C-NIC cells (*p* < 0.001, Figure 6A). Decreased levels of cytosolic LC3 were also accompanied by diminished pools of p62 and lysosome number in U-251 MG D-NIC cells upon drug stimulations (*p* < 0.001, Figure 6A). This may suggest that *DNMT2/TRDMT1* gene knockout resulted in impaired autophagic response in glioblastoma cells. It was reported that DOX-mediated autophagy may determine cellular context-dependent cancer cell fates [31, 42]. A blockage of apoptosis by pharmacological inhibitor ZVAD-fmk promoted cytotoxic autophagy in human breast cancer cells [31]. In contrast, in human osteosarcoma cells, DOX treatment stimulated cytoprotective autophagy that attenuated apoptotic cell death [42]. Genetic (ATG7 siRNA) or pharmacological (3-methyladenine) inhibition of autophagy potentiated DOX-induced apoptosis in osteosarcoma cells [42]. Except of AZA-mediated increase in p62 levels in DOX- and ETOPO-treated U-251 MG D-NIC cells, the effect of AZA on autophagy parameters was limited in cancer cells with *DNMT2/TRDMT1* gene knockout (Figure 6A and Supplementary Figure 7).

Low dose DOX treatment (50 ng/ml that is equivalent to 90 nM) resulted also in hepatoma cell death through mitotic catastrophe accompanied by senescence-like phenotype [43]. DOX-induced senescence was preceded by multinucleation and downregulation of mitotic checkpoint proteins such as CENP-A, Mad2, BubR1 and Chk1 [43]. As an increase in micronuclei production and multinucleated cells (polyploid giant cells) and polyploidy may be associated with chemotherapy resistance [44–46] and/or a selective form of autophagy, namely nucleophagy [47–49], we have then analyzed drug-mediated multinucleation and the effect of *DNMT2/TRDMT1* gene knockout (Figure 6B and Supplementary Figure 7). Indeed, DOX and ETOPO treatments resulted in increased levels of multinucleated cells in four cancer cell lines (*p* < 0.001, Figure 6B and Supplementary Figure 7). However, except of DOX-associated decrease in the levels of multinucleated cells in HeLa D-NIC cells compared to corresponding DOX-treated C-NIC cells (*p* < 0.001, Supplementary Figure 7A), *DNMT2/TRDMT1* gene knockout did not affect drug-mediated elevation in the levels of multinucleated cells (Figure 6B and Supplementary Figure 7). AZA also did not stimulate the occurrence of multinucleated cells (Figure 6B and Supplementary Figure 7). It was reported that chemo-resistance (DOX, paclitaxel, docetaxel) in multinucleated breast cancer cells is achieved by ROS-HIF-1α signaling axis [46]. Multinucleated breast cancer cells are characterized by elevated levels of ROS that stabilized HIF-1α and promoted VEGF and MIF secretion, which in turn upregulated anti-apoptotic proteins contributing to chemo-resistance [46].

### *DNMT2/TRDMT1* gene knockout modulates SASP during drug-induced senescence in glioblastoma cells

**Figure 7:**
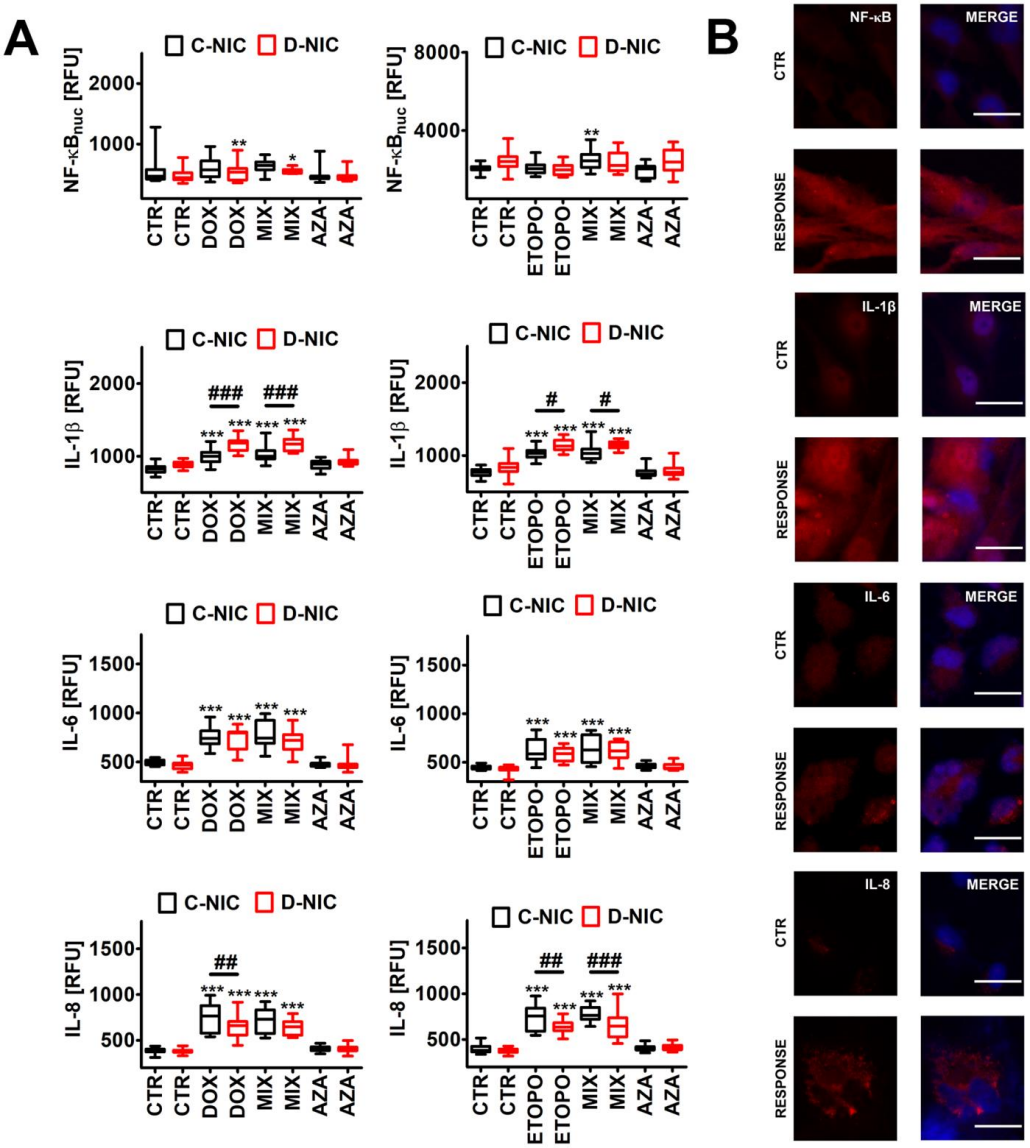
*DNMT2/TRDMT1* gene knockout-mediated senescence-associated secretory phenotype (SASP) (**A**, **B**) in U-251 MG glioblastoma cells treated with DOX or ETOPO. (**A**) The levels of NF-κB, IL-1β, IL-6 and IL-8 are expressed as relative fluorescence units (RFU). Box and whisker plots are shown, n = 3, ^***^p < 0.001, ^**^p < 0.01, ^*^p < 0.05 compared to CTR (ANOVA and Dunnett’s *a posteriori* test), ^###^*p* < 0.001, ^##^*p* < 0.01, ^#^*p* < 0.05 compared to C-NIC cells at the same culture conditions (ANOVA and Tukey’s *a posteriori* test). (**B**) NF-κB, IL-1β, IL-6 and IL-8 immunostaining (red). Representative microphotographs are shown, objective 20x, nucleus staining (blue), RESPONSE, representative DOX or ETOPO treatment. CTR, control conditions; DOX, doxorubicin treatment; ETOPO, etoposide treatment; AZA, azacytidine treatment; MIX, azacytidine post-treatment; C-NIC, control cells with unmodified levels of DNMT2/TRDMT1 containing control plasmid; D-NIC, cells with *DNMT2/TRDMT1* gene knockout containing dedicated DNMT2 double nickase plasmid.

As stress-induced premature senescence (SIPS), here DOX- and ETOPO-stimulated senescence in cancer cells, is accompanied by senescence-associated secretory phenotype (SASP) [1, 8, 9], we have then decided to evaluate the levels of nuclear NF-κB, a major regulator of proinflammatory response [6] and the levels of selected cytokines, namely IL-1β, IL-6 and IL-8 (Figure 7 and Supplementary Figure 8).

In general, the treatments with anti-cancer drugs did not substantially affect the levels of nuclear NF-κB (Figure 7 and Supplementary Figure 8), thus NF-κB might not be involved in the regulation of proinflammatory secretion of senescent cancer cells in our experimental settings. Perhaps other pathways may be implicated in DOX- and ETOPO-stimulated SASP (this study) [7, 8]. Indeed, DOX, ETOPO and 5-fluorouracil may activate p38 MAPK in murine macrophages and Lewis lung carcinoma (LLC1) cells [50]. Drug-induced p38 MAPK activity was associated with increased production of inflammatory cytokines IL-1β, TNF-α and IL-6 [50]. DOX and ETOPO also promoted an increase in the production of IL-1β, IL-6 and IL-8 in four cancer cell lines (*p* < 0.001, Figure 7A and Supplementary Figure 8). The effect of *DNMT2/TRDMT1* gene knockout on secretory profiles was the most evident in DOX- and ETOPO-treated U-251 MG D-NIC cells (Figure 7A). *DNMT2/TRDMT1* gene knockout resulted in increased production of IL-1β and decreased production of IL-8 in drug-treated U-251 MG D-NIC cells compared to corresponding drug-treated C-NIC cells (Figure 7A). DOX did not affect interleukin levels in HeLa, MDA-MB-231 and U-2 OS D-NIC cells compared to corresponding drug-treated C-NIC cells (Supplementary Figure 8A) and the effects of ETOPO treatment were limited to ETOPO-induced increase in the levels of IL-1β in U-2 OS D-NIC cells and IL-6 in HeLa D-NIC cells and decrease in the levels of IL-8 in MDA-MB-231 D-NIC cells (Supplementary Figure 8B). IL-1 signaling may contribute to acute DOX-induced cardiotoxicity, thus its inhibition may limit cardiomyocyte toxicity in DOX-exposed patients [51]. IL-1α also increased the sensitivity of MG-63, SAOS-2 and TE-85 osteosarcoma cells to etoposide when these two agents were used simultaneously [52]. ETOPO-treated U-2 OS D-NIC osteosarcoma cells were also characterized by elevated secretion of IL-1β that was accompanied by increased sensitivity to ETOPO stimulation compared to U-2 OS C-NIC cells (*p* < 0.001, Supplementary Figures 2, 8B). IL-1 signaling may also modulate tumor microenvironment and promote cancer initiation and progression [53]. Moreover, IL-1-mediated activation of anti-apoptotic signaling pathways and senescence may contribute to resistance to targeted therapies [53]. IL-1α and IL-1β signals through IL-1R may promote the SASP response in a cooperative manner that may trigger some protumorigenic effects [54]. Indeed, IL-1α inactivation impaired tumor progression and immune cell infiltration without affecting cell cycle arrest in a mouse model of pancreatic cancer [54]. AZA did not promote a proinflammatory response in four cancer cell lines (Figure 7 and Supplementary Figure 8).

### *DNMT2/TRDMT1* gene knockout potentiates drug-induced increase in the levels of NSUN6 in glioblastoma cells

**Figure 8:**
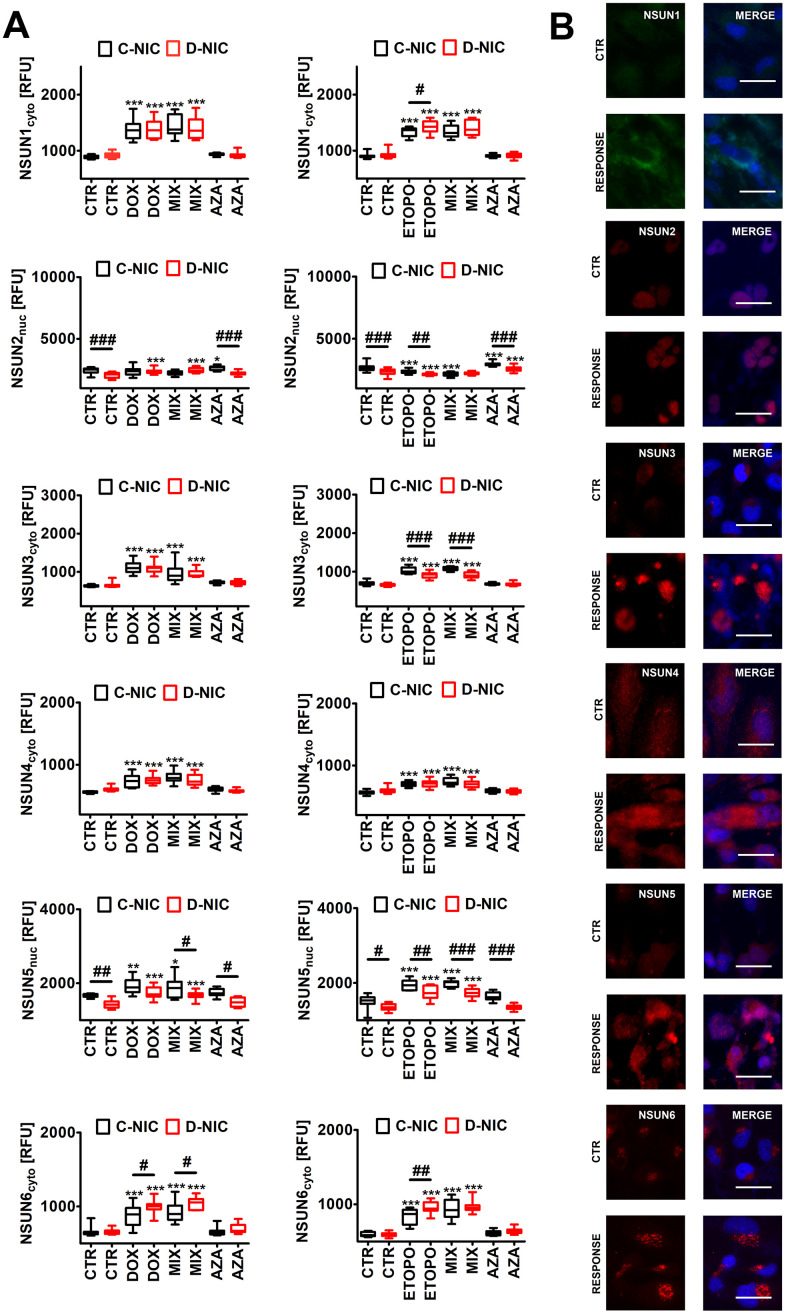
*DNMT2/TRDMT1* gene knockout-mediated changes in the levels of NSUN proteins (**A**, **B**) in U-251 MG glioblastoma cells treated with DOX or ETOPO. (**A**) The levels of NSUN1, NSUN2, NSUN3, NSUN4, NSUN5 and NSUN6 are expressed as relative fluorescence units (RFU). Box and whisker plots are shown, n = 3, ^***^p < 0.001, ^**^p < 0.01, ^*^p < 0.05 compared to CTR (ANOVA and Dunnett’s *a posteriori* test), ^###^*p* < 0.001, ^##^*p* < 0.01, ^#^*p* < 0.05 compared to C-NIC cells at the same culture conditions (ANOVA and Tukey’s *a posteriori* test). (**B**) NSUN1, NSUN2, NSUN3, NSUN4, NSUN5 and NSUN6 immunostaining (red). Representative microphotographs are shown, objective 20x, nucleus staining (blue), RESPONSE, representative DOX or ETOPO treatment. CTR, control conditions; DOX, doxorubicin treatment; ETOPO, etoposide treatment; AZA, azacytidine treatment; MIX, azacytidine post-treatment; C-NIC, control cells with unmodified levels of DNMT2/TRDMT1 containing control plasmid; D-NIC, cells with *DNMT2/TRDMT1* gene knockout containing dedicated DNMT2 double nickase plasmid.

We were then interested if *DNMT2/TRDMT1* gene knockout may modulate the levels of other RNA methyltransferases, namely the NOL1/NOP2/SUN domain (NSUN) family proteins NSUN1, NSUN2, NSUN3, NSUN4, NSUN5 and NSUN6 (Figure 8 and Supplementary Figure 9).

Surprisingly, except of the levels of NSUN2, the pools of NSUN proteins were elevated during DOX- and ETOPO-mediated senescence in four cancer cell lines (*p* < 0.001, Figure 8A and Supplementary Figure 9). In general, *DNMT2/TRDMT1* gene knockout did not augment the levels of NSUN proteins after drug treatment (Figure 8 and Supplementary Figure 9). One exception was the elevation of NSUN6 levels in DOX- and ETOPO-treated U-251 MG D-NIC cells compared to corresponding drug-treated C-NIC cells (*p* < 0.05 and *p* < 0.01, Figure 8A). AZA did not affect the levels of NSUN proteins in four cancer cell lines (Figure 8 and Supplementary Figure 9). There are conflicting reports on the complex role(s) of NSUN proteins in cancer biology and cellular senescence [55–57]. For example, NSUN2 may both delay and stimulate cellular senescence that may reflect different cellular contexts [56, 57]. NSUN proteins may also exert both cancer promoting and inhibiting effects [58–61]. NSUN6 may suppress the proliferation and development of liver and pancreatic cancer cells [60, 61]. However, in our experimental conditions, increased levels of NSUN6 in DOX- and ETOPO-treated U-251 MG D-NIC cells were accompanied by apoptosis resistance compared to corresponding C-NIC cells (Figures 3, 8).

### *DNMT2/TRDMT1* gene knockout lowers the cytoplasmic pools of 5-methylcytosine during drug-induced senescence in glioblastoma cells

We have then analyzed if the lack of active *DNMT2/TRDMT1* gene may affect an epigenetic mark, namely the levels of nuclear and cytoplasmic fractions of 5-methylcytosine. DOX and ETOPO treatments resulted in increased levels of both nuclear and cytoplasmic pools of 5-methylcytosine in four cancer cell lines (Supplementary Figure 10). *DNMT2/TRDMT1* gene knockout-associated decrease in cytoplasmic fraction of 5-methylcytosine was only observed in DOX- and ETOPO-treated U-251 MG D-NIC cells, ETOPO-treated HeLa D-NIC cells and DOX-treated U-2 OS D-NIC cells compared to corresponding drug-treated C-NIC cells (*p* < 0.05 and *p* < 0.001, Supplementary Figure 10). As the levels of NSUN proteins were increased during DOX- and ETOPO-induced senescence in four cancer cell lines (*p* < 0.001, Figure 8A and Supplementary Figure 9), one can speculate that limited impact of *DNMT2/TRDMT1* gene knockout on the cytoplasmic pools of 5-methylcytosine is based on a compensation effect of elevated levels of NSUN proteins. However, we analyzed immunofluorescence-based changes in global 5-methylcytosine that did not allow for the discrimination between methylation patterns of different types of RNAs. AZA did not substantially affect the levels of nuclear and cytoplasmic 5-methylcytosine in four cancer cell lines (Supplementary Figure 10).

**Figure 9:**
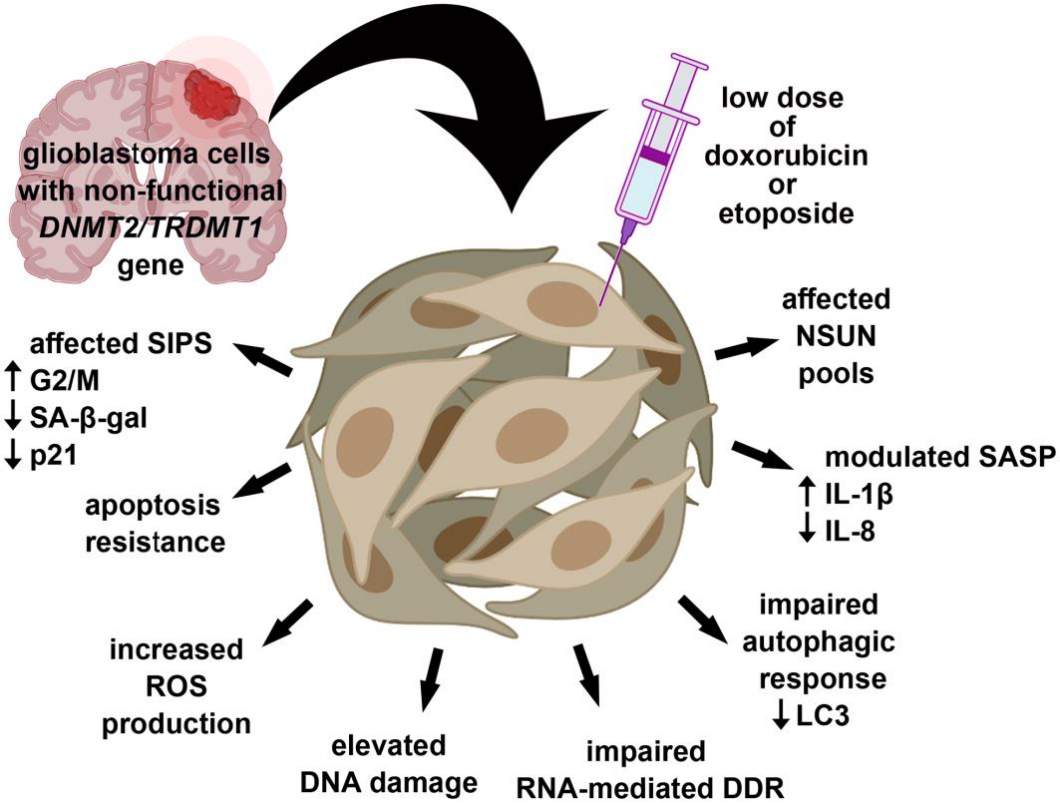
***DNMT2/TRDMT1* gene knockout modulated DOX- and ETOPO-induced senescence program in glioblastoma cells as judged by affected SIPS response (lowered number of SA-beta-gal-positive cells and diminished levels of nuclear p21), apoptosis resistance, increased ROS production, increased DSBs, impaired RNA-mediated DDR and autophagic response, modulated SASP and the levels of NSUN proteins.** Thus, *DNMT2/TRDMT1* gene knockout may result in the promotion of some selected adverse side effects mediated by drug-stimulated senescence.

In conclusion, we have shown for the first time that *DNMT2/TRDMT1* gene knockout affected DOX- and ETOPO-mediated senescence program in glioblastoma cells as judged by modulated SIPS response (decreased number of SA-beta-gal-positive cells and nuclear p21 pools), apoptosis resistance, increased ROS levels, elevated DNA damage and impaired RNA-mediated DDR, limited autophagic response, modulated SASP and affected NSUN levels (Figure 9).

Thus, the lack of functional *DNMT2/TRDMT1* gene in glioblastoma cells may potentiate some adverse side effects associated with chemotherapy-induced senescence. A summary of obtained results using four different cancer cell lines, namely HeLa, MDA-MB-231, U-2 OS and U-251 MG cells is also presented in Table 1.

**Table 1:** A comparison between analyzed parameters in cells lacking active *DNMT2/TRDMT1* gene (D-NIC cells) and in cells with functional *DNMT2/TRDMT1* gene (C-NIC cells) during doxorubicin- or etoposide-induced senescence (35 nM DOX or 1 μM ETOPO).

**Parameter**	**C-NIC cells versus D-NIC cells**							
	**HeLa**		**MDA-MB-231**		**U-2 OS**		**U-251 MG**	
	**DOX**	**ETOPO**	**DOX**	**ETOPO**	**DOX**	**ETOPO**	**DOX**	**ETOPO**
**cell cycle**	**-**	**G0/G1↑**	**G2/M↑**	**G2/M↑**	**-**	**-**	**G2/M↑**	**G2/M↑**
**SA-β-gal activity**	**-**	**-**	**-**	**-**	**-**	**-**	**↓**	**↓**
**p21**	**-**	**-**	**↓**	**↓**	**-**	**-**	**↓**	**↓**
**apoptosis**	**↑**	**-**	**↑**	**↑**	**↓**	**↑**	**↓**	**-**
**senolytic activity of 1 μM AZA**	**↑** **(apoptosis)**	**↑** **(apoptosis)**	**-**	**-**	**-**	**↑** **(necrosis)**	**-**	**-**
**ROS**	**↑**	**-**	**-**	**↑**	**↓**	**↓**	**↑**	**↑**
**DNA damage**	**-**	**↓**	**↑**	**-**	**-**	**↓**	**↑**	**-**
**micronuclei**	**-**	**↓**	**-**	**-**	**-**	**-**	**↓**	**↓**
**pATM(+)pH2AX(+)**	**↓**	**↓**	**-**	**↓**	**↑**	**↑**	**↓**	**-**
**53BP1**	**↑**	**↑**	**↑**	**-**	**-**	**↓**	**↓**	**-**
**RNA-DNA hybrids**	**-**	**-**	**-**	**-**	**-**	**-**	**↓**	**↓**
**RAD51**	**↑**	**-**	**-**	**-**	**-**	**-**	**↓**	**↓**
**RAD52**	**-**	**-**	**-**	**-**	**-**	**↓**	**↓**	**↓**
**XRCC1**	**↑**	**-**	**-**	**↓**	**↓**	**↓**	**↑**	**↑**
**LC3**	**-**	**↓**	**-**	**↓**	**-**	**-**	**↓**	**↓**
**p62**	**↑**	**↑**	**-**	**↓**	**-**	**↑**	**-**	**-**
**lysosomes**	**↑**	**↑**	**-**	**-**	**↑**	**↑**	**↓**	**-**
**NF-κB**	**↑**	**-**	**-**	**-**	**-**	**↓**	**-**	**-**
**IL-1β**	**-**	**-**	**-**	**-**	**-**	**↑**	**↑**	**↑**
**IL-6**	**-**	**↑**	**-**	**-**	**-**	**-**	**-**	**-**
**IL-8**	**-**	**-**	**-**	**↓**	**-**	**-**	**↓**	**↓**
**NSUN1**	**↓**	**-**	**↓**	**-**	**-**	**-**	**-**	**↑**
**NSUN2**	**-**	**-**	**↑**	**↑**	**↓**	**↓**	**-**	**↓**
**NSUN3**	**-**	**-**	**↓**	**-**	**-**	**↑**	**-**	**↓**
**NSUN4**	**-**	**-**	**↓**	**-**	**-**	**-**	**-**	**-**
**NSUN5**	**-**	**-**	**-**	**-**	**-**	**-**	**-**	**↓**
**NSUN6**	**-**	**-**	**-**	**-**	**-**	**-**	**↑**	**↑**
**cytoplasmic 5-mC**	**↑**	**↓**	**-**	**-**	**↓**	**-**	**↓**	**↓**
**nuclear 5-mC**	**-**	**-**	**-**	**↑**	**↓**	**-**	**-**	**↓**

Four different cancer cellular models were considered, namely HeLa, MDA-MB-231, U-2 OS and U-251 MG cells. ↑ (red), statistically significant increase in analyzed parameter compared to corresponding C-NIC cells; ↓ (green), statistically significant decrease in analyzed parameter compared to corresponding C-NIC cells; - (gray), no statistically significant changes compared to corresponding C-NIC cells.

As one can observe in Table 1, *DNMT2/TRDMT1* gene knockout-associated responses during drug-induced senescence may depend on cellular context.

## MATERIALS AND METHODS

### Cancer cell lines and transfection protocol

Four human cancer cell lines originated from different tissues were used, namely MDA-MB-231 breast cancer cells (ATCC^®^ HTB-26^™^, ATCC, Manassas, VA, USA), HeLa cervical cancer cells (ATCC^®^ CCL-2^™^, ATCC, Manassas, VA, USA), U-2 OS osteosarcoma cells (92022711, ECACC, Public Health England, Porton Down, Salisbury, UK) and U-251 MG glioblastoma cells (09063001, ECACC, Public Health England, Porton Down, Salisbury, UK). Cells were routinely cultured at 37° C in DMEM medium supplemented with 10% FBS and 100 U/ml penicillin, 0.1 mg/ml streptomycin, and 0.25 μg/ml amphotericin B (Corning, Tewksbury, MA, USA) in the presence of 5% CO_2_. CRISPR-based technology was considered to knockout the *DNMT2*/*TRDMT1* gene. Briefly, cells were transfected with DNMT2 Double Nickase Plasmids (h, sc-402709-NIC and h2, sc-402709-NIC-2, Santa Cruz Biotechnology, Dallas, TX, USA) using Lipofectamine^™^ 3000 (Thermo Fisher Scientific, Waltham, MA, USA) according to the manufacturer’s instructions. Plasmid-related effects (control cells, C-NIC cells) were also analyzed using Control Double Nickase Plasmid (sc-437281, Santa Cruz Biotechnology, Dallas, TX, USA). Cells with *DNMT2/TRDMT1* gene knockout (D-NIC cells) were selected upon puromycin treatment (sc-108071, Santa Cruz Biotechnology, Dallas, TX, USA) that was confirmed using anti-DNMT2 antibody (A-7, sc-271513, Santa Cruz Biotechnology, Dallas, TX, USA) and Western blotting protocol [21].

### Anti-cancer drug treatment

Three anti-cancer drugs, namely doxorubicin hydrochloride (DOX, 44583), etoposide (ETOPO, E1383) and 5-azacytidine (AZA, A2385) were obtained from Merck KGaA (Darmstadt, Germany). To induce drug-mediated senescence, C-NIC and D-NIC cells were treated with 35 nM DOX or 1 μM ETOPO for 24 h and then cultured without drugs for 7 days. Moreover, the action of AZA, a hypomethylating agent, was also studied in D-NIC cells lacking active *DNMT2/TRDMT1* gene to analyze combined effects of the inhibition of RNA and DNA methylation. Briefly, C-NIC and D-NIC cells were treated with 1 μM AZA for 24 h after 7 days of DOX or ETOPO removal. Moreover, DOX-treated or ETOPO-treated cells for 24 h were stimulated with 1 μM AZA for additional 24 h and culture was then terminated. If not indicated otherwise, the parameters were investigated after 7 days of drug removal (DOX or ETOPO) and 24 h treatment with AZA.

### Cell cycle analysis

C-NIC and D-NIC cells were treated with 35 nM DOX or 1 μM ETOPO for 24 h and/or with 1 μM AZA for additional 24 h and drug-mediated changes in the phases of the cell cycle (G0/G1, S, and G2/M) were then revealed using a Muse^®^ Cell Analyzer and a Muse^®^ Cell Cycle Assay Kit according to the manufacturer’s instructions (Luminex Corporation, Austin, TX, USA).

### Senescence-associated β-galactosidase activity

Cellular senescence was evaluated using CellEvent^™^ Senescence Green Detection Kit according to the manufacturer’s instructions (Thermo Fisher Scientific, Waltham, MA, USA). Briefly, upon induction of cellular senescence using anti-cancer drugs and a 96 well plate format culture, C-NIC and D-NIC cells were fixed, washed and stained according to the supplier’s protocol. Digital cell images were captured using a laser-based confocal imaging and high content analysis (HCA) system IN Cell Analyzer 6500 HS (Cytiva, Marlborough, MA, USA). Quantitative analysis was conducted using IN Carta software (Cytiva, Marlborough, MA, USA). Senescence-associated β-galactosidase activity is presented as relative fluorescence units (RFU).

### Apoptosis and necrosis

C-NIC and D-NIC cells were treated with 35 nM DOX or 1 μM ETOPO for 24 h and/or with 1 μM AZA for additional 24 h and drug-mediated apoptosis was then evaluated using a Muse^®^ Cell Analyzer and Muse^®^ Annexin V and Dead Cell Assay Kit (phosphatidylserine externalization) according to manufacturer’s instructions (Luminex Corporation, Austin, TX, USA). Four cell subpopulations were documented, namely (1) Annexin V (−) and 7-AAD (−), (2) Annexin V (+) and 7-AAD (−), (3) Annexin V (+) and 7-AAD (+), and (4) Annexin V (−) and 7-AAD (+) (%). Representative dot plots are shown. Apoptosis-based or necrosis-based senolytic activity of AZA was also analyzed upon 7 days of drug removal (DOX or ETOPO) and 24 h stimulation with AZA.

### Superoxide levels

C-NIC and D-NIC cells were treated with 35 nM DOX or 1 μM ETOPO for 24 h and/or with 1 μM AZA for additional 24 h and drug-mediated changes in intracellular superoxide levels were analyzed using Muse^®^ Cell Analyzer and Muse^®^ Oxidative Stress Kit (Luminex Corporation, Austin, TX, USA) [62]. Two subpopulations were considered, namely, superoxide-positive and superoxide-negative cells, and representative histograms are presented (%).

### Immunofluorescence and GFP-based imaging

Cells were fixed and immuno-stained as comprehensively described elsewhere [63]. The following primary and secondary antibodies were considered, namely, anti-p21 (1:800, MA5-14949), anti-53BP1 (1:1000, PA1-16565), anti-RAD51 (1:100, MA5-14419), anti-RAD52 (1:500, MA5-31888), anti-XRCC1 (1:200, MA5-12071), anti-RNA-DNA hybrid (1:100, MABE1095), anti-NF-κB (p65) (1:100, PA5-16545), anti-IL-1β (1:200, P420B), anti-IL-6 (1:500, ab6672), anti-IL-8 (1:500, ab154390), anti-LC3B (1:500, PA5-32254), anti-NSUN1/NOP2 (1:500, ab4638), anti-NSUN2 (1:250, 702036), anti-NSUN3 (1:200, PA5-57561), anti-NSUN4 (1:250, 720212), anti-NSUN5 (1:200, PA5-54228), anti-NSUN6 (1:600, PA5-61119), anti-5-mC (1:140, ab51552), anti-Ago2 (1:500, MA5-23515), and secondary antibodies conjugated to Texas Red (1:1000, T2767) or Alexa Fluor Plus 488 (1:1000, A32723) or Texas Red-X (1:1000, T6390) (Thermo Fisher Scientific, Waltham, MA, USA, Merck KGaA, Darmstadt, Germany and Abcam, Cambridge, UK). Fixed cells were incubated with primary antibodies at 4° C overnight and secondary antibodies at room temperature for 1 h. Nuclei were visualized using Hoechst 33342 staining. Digital cell images were captured using a laser-based confocal imaging and HCA system IN Cell Analyzer 6500 HS (Cytiva, Marlborough, MA, USA). Quantitative analysis was conducted using IN Carta software (Cytiva, Marlborough, MA, USA). The immuno-fluorescent signals of analyzed proteins (protein levels) are presented as relative fluorescence units (RFU). If applicable, a cellular localization is provided. 53BP1 and RNA-DNA hybrid foci were calculated per cell.

GFP-based imaging of a lysosomal marker Lamp1 and an autophagy marker p62 (SQSTM1) was conducted using CellLight^™^ Lysosomes-GFP, BacMam 2.0 and Premo^™^ Autophagy Sensor GFP-p62 Kit (C10596 and P36240, Thermo Fisher Scientific, Waltham, MA, USA) according to the manufacturer’s instructions, respectively. GFP-Lamp1 or GFP-p62 were transduced into C-NIC and D-NIC cells *via* baculovirus-based system (BacMam 2.0 technology), and GFP-Lamp1 or GFP-p62 fluorescent signals were captured using a laser-based confocal imaging and HCA system IN Cell Analyzer 6500 HS (Cytiva, Marlborough, MA, USA). Quantitative analysis was conducted using IN Carta software (Cytiva, Marlborough, MA, USA). The fluorescence signals of analyzed proteins (protein levels) are presented as relative fluorescence units (RFU).

### Genetic instability, DNA damage and DNA damage response (DDR)

Micronuclei (MN) formation and multinucleation events were evaluated using Hoechst 33342 staining and digital cell images were captured using a laser-based confocal imaging and HCA system IN Cell Analyzer 6500 HS (Cytiva, Marlborough, MA, USA). Quantitative analysis was conducted using IN Carta software (Cytiva, Marlborough, MA, USA). Micronuclei production and multinucleation events were expressed as %.

DNA double-strands breaks (DSBs) were assessed using neutral comet assay. Briefly, C-NIC and D-NIC cells (10^5^/ml) were combined with 1% low melting agarose in PBS at a ratio of 1:10 (v/v) and 50 μl were pipetted onto a CometSlide^™^ (Trevigen, Gaithersburg, MD, USA). The slides were lysed using a lysis solution (10 mM Tris-HCl, 2.5 M NaCl, 100 mM EDTA, 1% N-lauroylsarcosine, 10% DMSO, pH 10) at 4° C for 1 h. The slides were then incubated with an electrophoresis buffer (300 mM sodium acetate, 100 mM Tris-HCl, pH 10) for 20 min and electrophoresis (0.5 V/cm) was processed for 10 min (300 mM sodium acetate, 100 mM Tris-HCl, pH 10). The slides were then washed three times using a neutralization buffer (20 mM Tris-HCl, 50% EtOH, 1 mg/ml spermine, pH 7.5) for 30 min. The slides were then stained using a staining solution (0.25 μM YOYO-1, 2.5% DMSO, 0.5% sucrose, Thermo Fisher Scientific, Waltham, MA, USA). Digital comet images were captured using an Olympus BX61 fluorescence microscope equipped with a DP72 CCD camera and Olympus CellF software. The CCD capture conditions were: exposure time 100 ms, magnification 20×. DSBs as tail DNA (%) were analyzed using a OpenComet: an automated tool for comet assay image analysis [64].

The phosphorylation status of ATM and H2AX was assessed using Muse^®^ Cell Analyzer and Muse^®^ Multi-Color DNA Damage kit according to the manufacturer’s instructions (Luminex Corporation, Austin, TX, USA). The activation of ATM and H2AX was studied using a phospho-specific ATM (Ser1981)-PE and a phospho-specific histone H2AX-PECy5 conjugated antibodies. Four cell subpopulations were revealed using flow cytometry analysis, namely pATM(-) and pH2AX(-) cells (no DNA damage), pATM(+) and pH2AX(-) cells (ATM activation), pATM(-) and pH2AX(+) cells (H2AX activation) and pATM(+) and pH2AX(+) cells (dual activation of ATM and H2AX – cells with DSBs).

### Statistical analysis

The results represent the mean ± SD from at least three independent experiments. Box and whisker plots with median, lowest, and highest values were also used. Differences between control samples and treated samples were analyzed using one-way ANOVA and Dunnett’s multiple comparison test, whereas differences between C-NIC cells and D-NIC cells were revealed using one-way ANOVA and Tukey’s multiple comparison test. Statistical significance was evaluated using GraphPad Prism 5. *P*-values of less than 0.05 were considered significant.

## Supplementary Material

Supplementary Figures
