# Supplementary material for: The lack of functional DNMT2/TRDMT1 gene modulates cancer cell responses during drug-induced senescence
Source: Aging (Albany NY). 2021 Jun 17;13(12):15833–74. doi: 10.18632/aging.203203 (PMC8266355; doi:10.18632/aging.203203)
Supplement: Supplementary Figures [file aging-13-203203-s001.pdf]

# SUPPLEMENTARY FIGURES

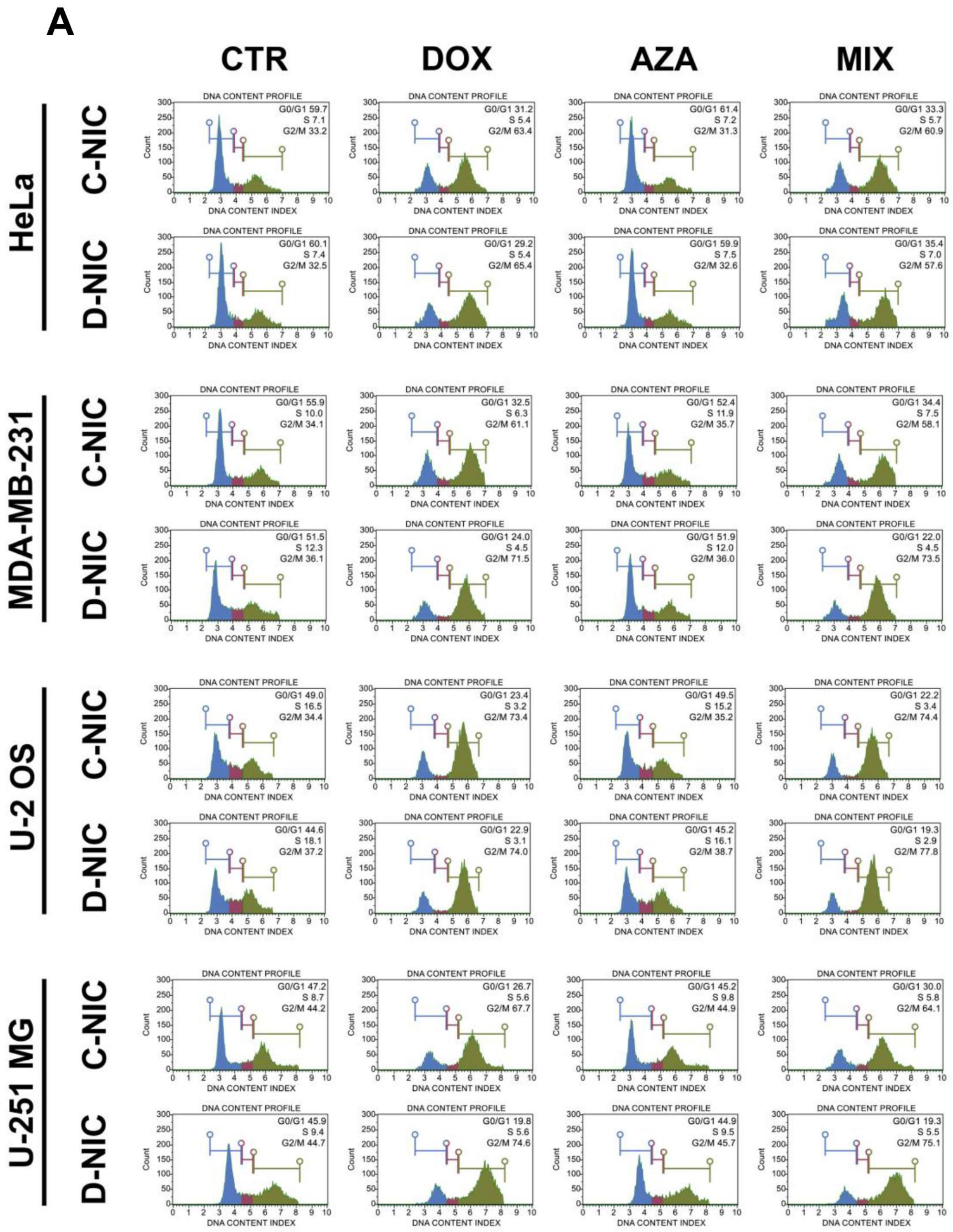

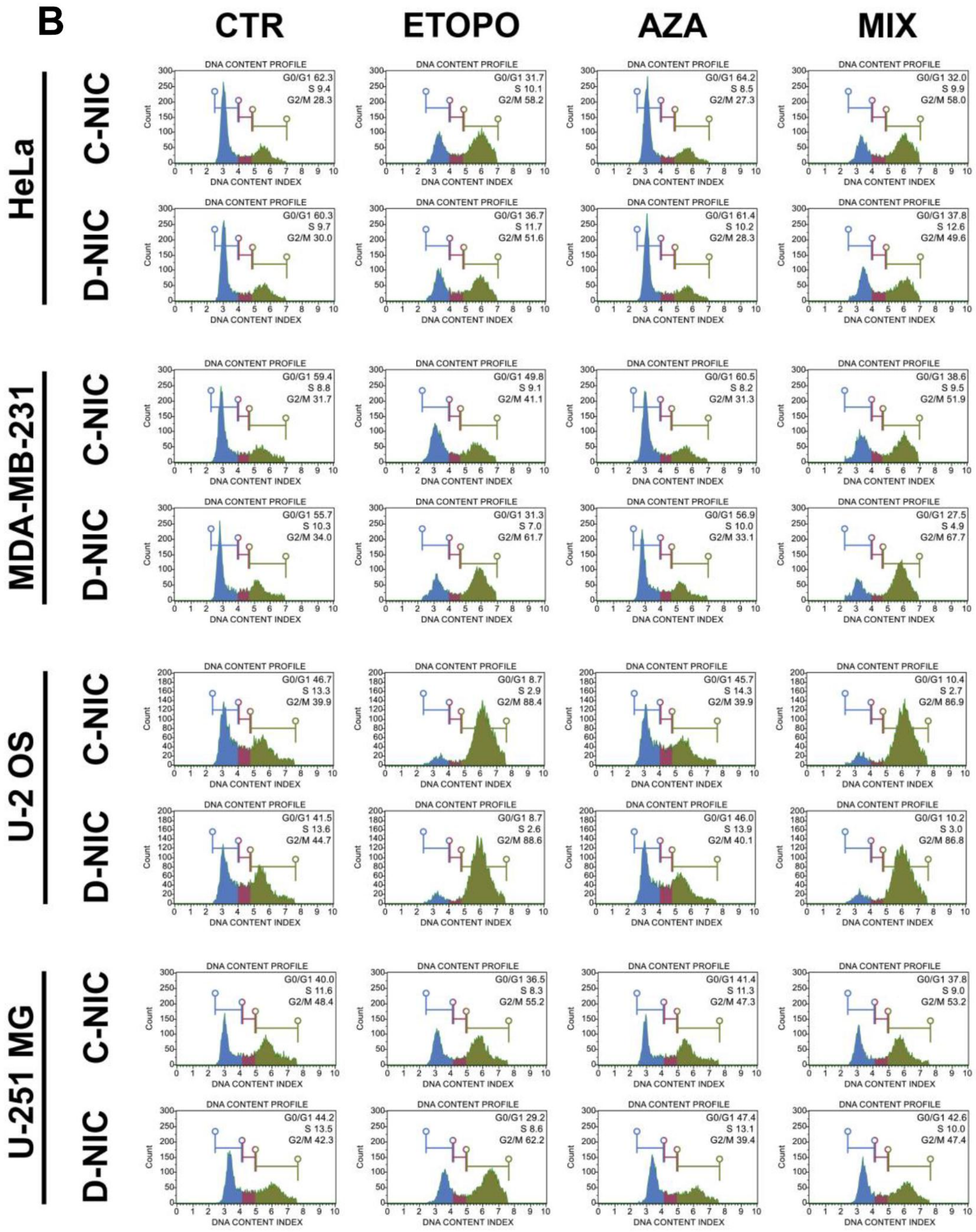


**Supplementary Figure 1.** *DNMT2/TRDMT1* gene knockout-mediated changes in the cell cycle of DOX- (**A**) and ETOPO-treated (**B**) cancer cells, namely HeLa cervical cancer cells, MDA-MB-231 breast cancer cells, U-2 OS osteosarcoma cells and U-251 MG glioblastoma cells. DNA content-based analysis of cell cycle was conducted using flow cytometry. Representative histograms are shown. CTR, control conditions; DOX, doxorubicin treatment; ETOPO, etoposide treatment; AZA, azacytidine treatment; MIX, azacytidine post-treatment; C-NIC, control cells with unmodified levels of DNMT2/TRDMT1 containing control plasmid; D-NIC, cells with *DNMT2/TRDMT1* gene knockout containing dedicated DNMT2 double nickase plasmid.

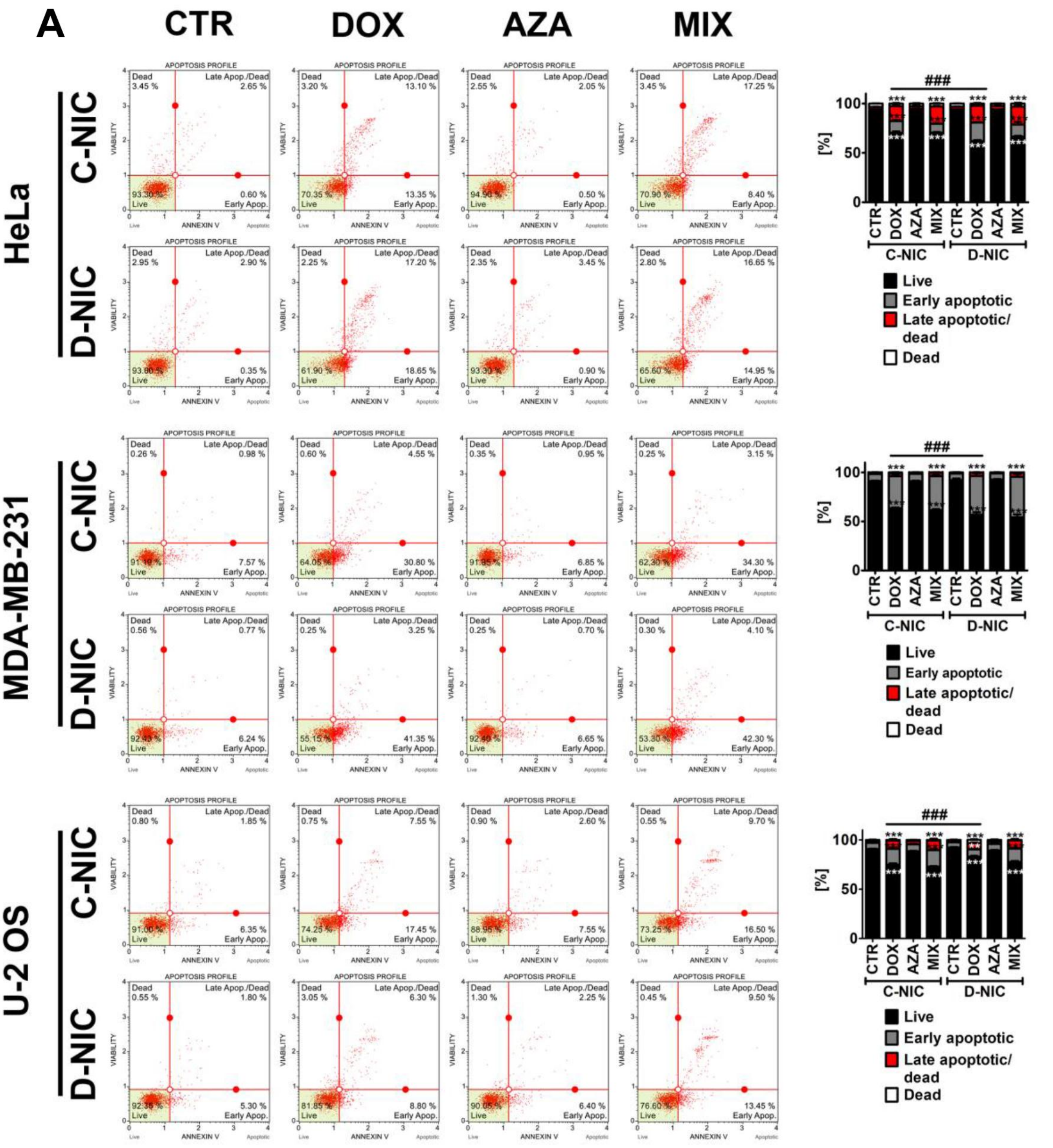
A
CTR
DOX
AZA
MIX
HeLa
C-NIC
D-NIC
MDA-MB-231
U-2 OS
APOPTOSIS PROFILE
ANNEXIN V
VIABILITY
[%]
###
Live
Early apoptotic
Late apoptotic/ dead
Dead

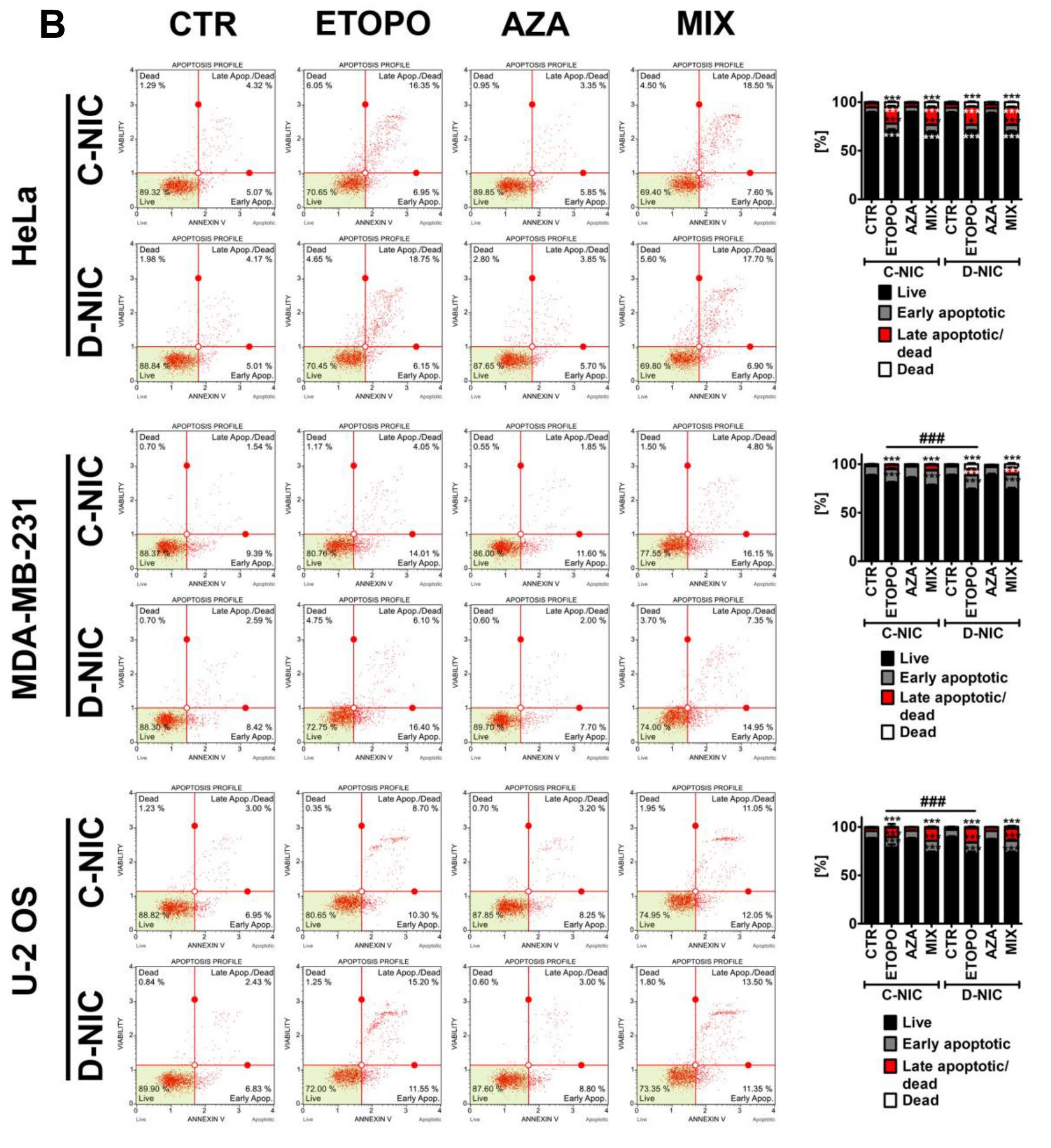


**Supplementary Figure 2.** *DNMT2/TRDMT1* gene knockout-mediated apoptosis and necrosis in three cancer cell lines, namely HeLa cervical cancer cells, MDA-MB-231 breast cancer cells and U-2 OS osteosarcoma cells treated with DOX (**A**) or ETOPO (**B**) for 24 h and immediately assayed. Apoptosis and necrosis were analyzed using flow cytometry. Representative dot plots are shown. Bars indicate SD, n = 3, $^{***}p < 0.001$, $^{**}p < 0.01$, $^{*}p < 0.05$ compared to CTR (ANOVA and Dunnett's *a posteriori* test), $^{\#\#\#}p < 0.001$ compared to drug-treated C-NIC cells (ANOVA and Tukey's *a posteriori* test). CTR, control conditions; DOX, doxorubicin treatment; ETOPO, etoposide treatment; AZA, azacytidine treatment; MIX, azacytidine post-treatment; C-NIC, control cells with unmodified levels of DNMT2/TRDMT1 containing control plasmid; D-NIC, cells with *DNMT2/TRDMT1* gene knockout containing dedicated DNMT2 double nickase plasmid.

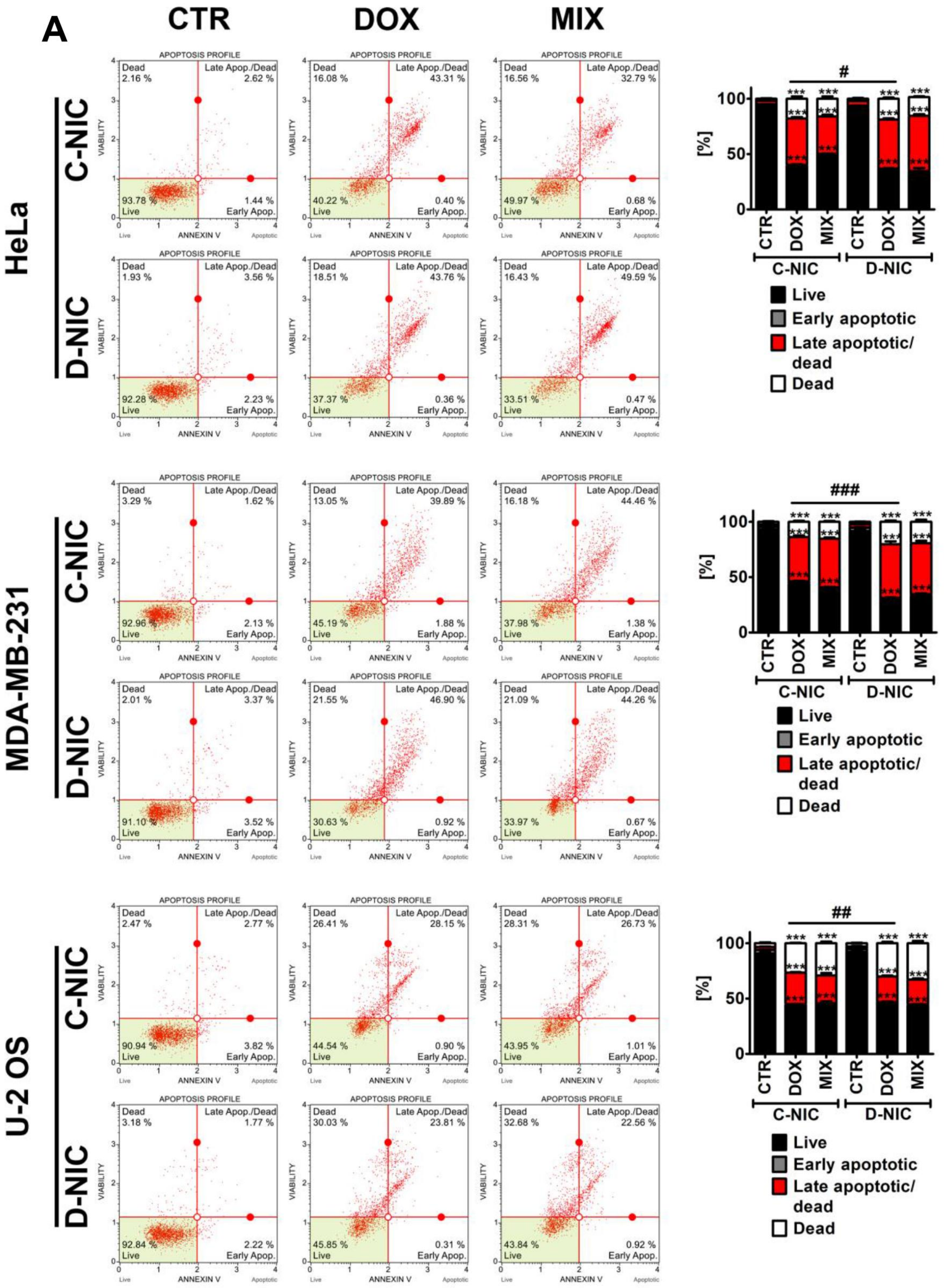
A
CTR
DOX
MIX
HeLa
MDA-MB-231
U-2 OS
C-NIC
D-NIC
APOPTOSIS PROFILE
VIABILITY
ANNEXIN V
Dead
Late Apop./Dead
Live
Early Apop.
Apoptotic
2.16 %
2.62 %
93.78 %
1.44 %
16.08 %
43.31 %
40.22 %
0.40 %
16.56 %
32.79 %
49.97 %
0.68 %
1.93 %
3.56 %
92.28 %
2.23 %
18.51 %
43.76 %
37.37 %
0.36 %
16.43 %
49.59 %
33.51 %
0.47 %
3.29 %
1.62 %
92.96 %
2.13 %
13.05 %
39.89 %
45.19 %
1.88 %
16.18 %
44.46 %
37.98 %
1.38 %
2.01 %
3.37 %
91.10 %
3.52 %
21.55 %
46.90 %
30.63 %
0.92 %
21.09 %
44.26 %
33.97 %
0.67 %
2.47 %
2.77 %
90.94 %
3.82 %
26.41 %
28.15 %
44.54 %
0.90 %
28.31 %
26.73 %
43.95 %
1.01 %
3.18 %
1.77 %
92.84 %
2.22 %
30.03 %
23.81 %
45.85 %
0.31 %
32.68 %
22.56 %
43.84 %
0.92 %
[%]
#
###
##
***
Live
Early apoptotic
Late apoptotic/dead
Dead

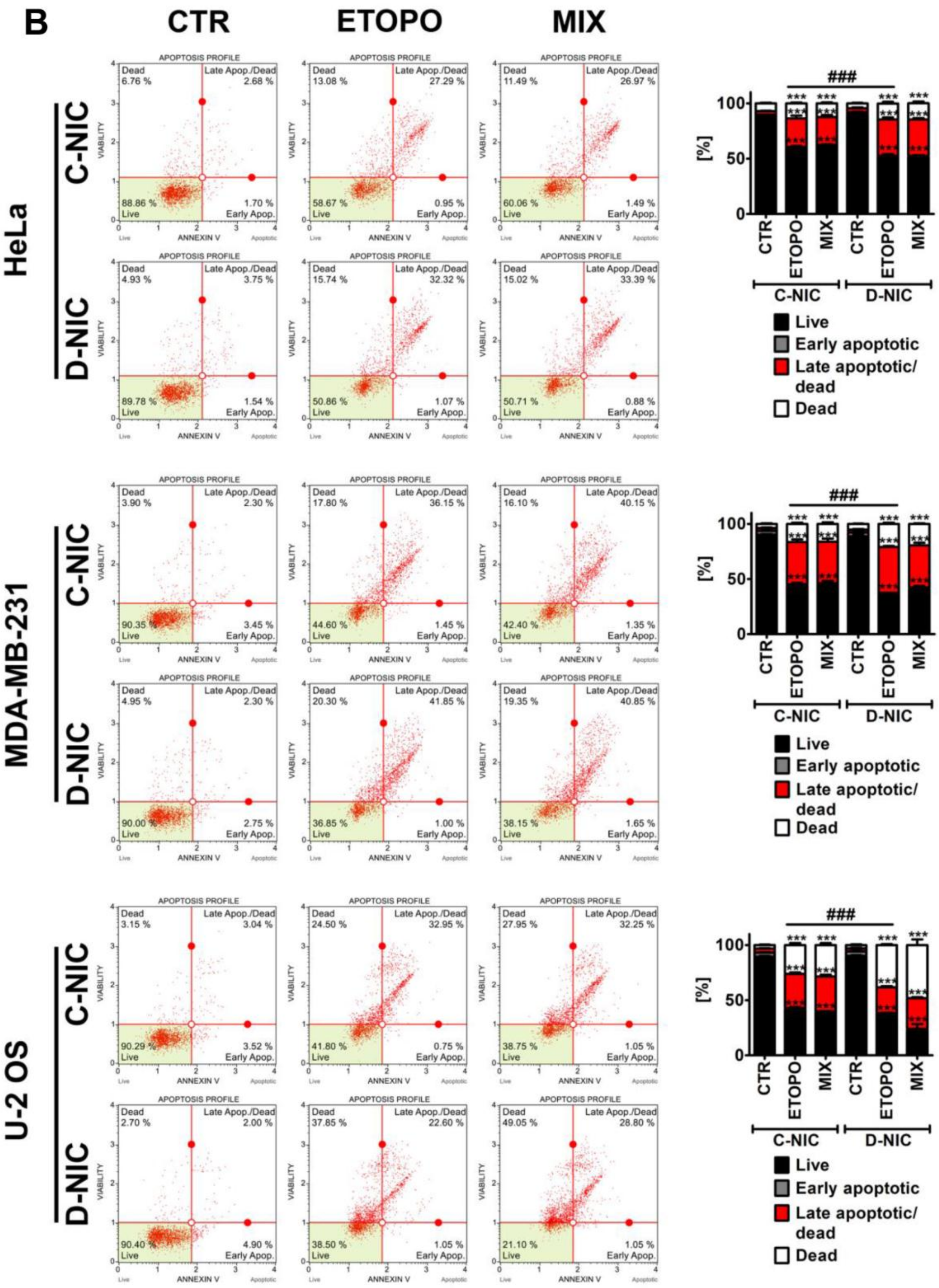


**Supplementary Figure 3.** *DNMT2/TRDMT1* gene knockout-mediated apoptosis and necrosis in three cancer cell lines, namely HeLa cervical cancer cells, MDA-MB-231 breast cancer cells and U-2 OS osteosarcoma cells treated with DOX (**A**) or ETOPO (**B**) for 24 h and assayed after 7 days of drug removal and AZA post-treatment for 24 h. Apoptosis and necrosis were analyzed using flow cytometry. Representative dot plots are shown. Bars indicate SD, n = 3, $^{***}p < 0.001$ compared to CTR (ANOVA and Dunnett's *a posteriori* test), $^{\#\#\#}p < 0.001$, $^{\#\#}p < 0.01$ $^{\#}p < 0.05$ compared to drug-treated C-NIC cells (ANOVA and Tukey's *a posteriori* test). CTR, control conditions; DOX, doxorubicin treatment; ETOPO, etoposide treatment; AZA, azacytidine treatment; MIX, azacytidine post-treatment; C-NIC, control cells with unmodified levels of DNMT2/TRDMT1 containing control plasmid; D-NIC, cells with *DNMT2/TRDMT1* gene knockout containing dedicated DNMT2 double nickase plasmid.

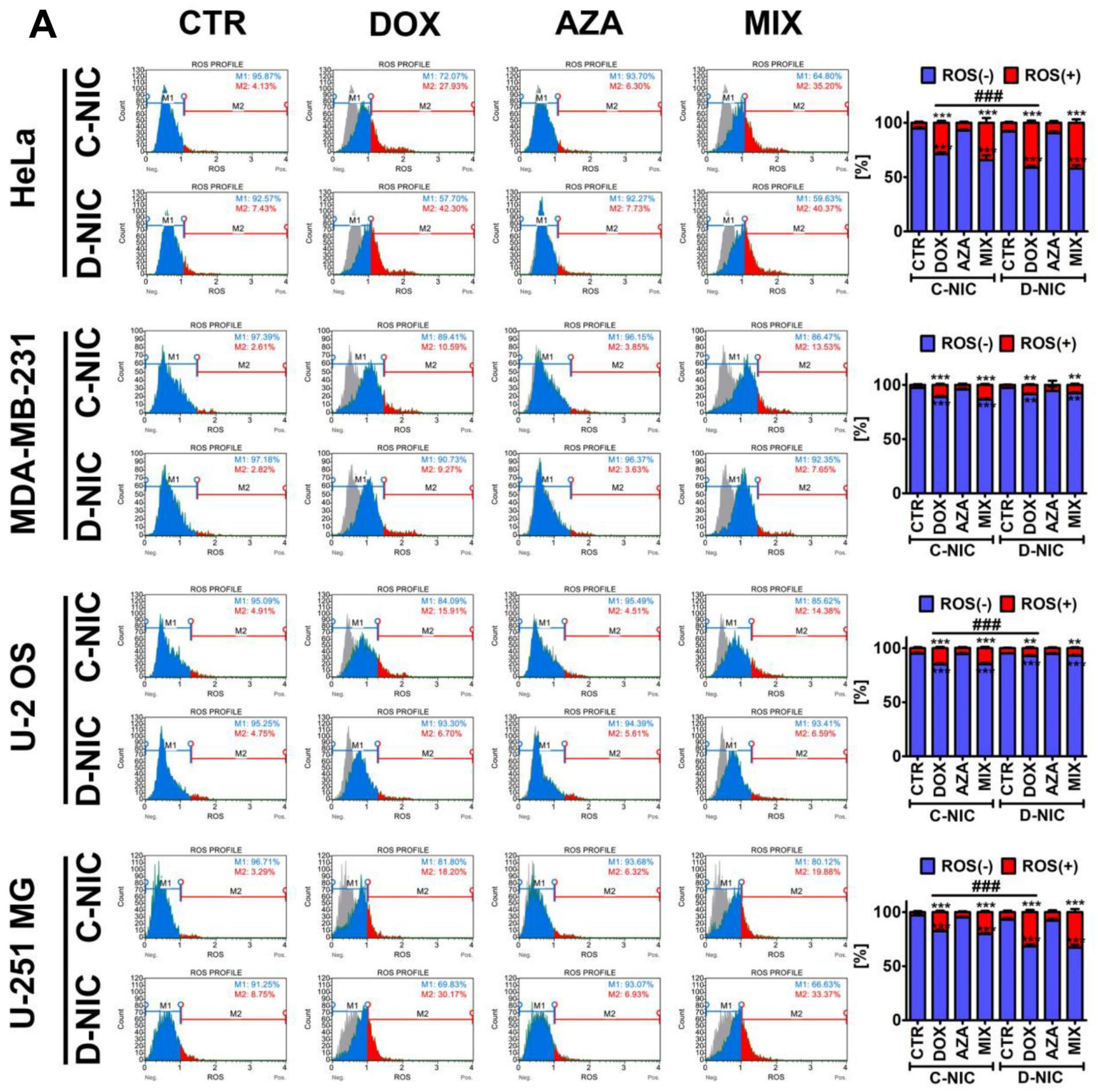
A
CTR
DOX
AZA
MIX
HeLa
C-NIC
D-NIC
ROS PROFILE
M1: 95.87% M2: 4.13%
M1: 72.07% M2: 27.93%
M1: 93.70% M2: 6.30%
M1: 64.80% M2: 35.20%
M1: 92.57% M2: 7.43%
M1: 57.70% M2: 42.30%
M1: 92.27% M2: 7.73%
M1: 59.63% M2: 40.37%
MDA-MB-231
M1: 97.39% M2: 2.61%
M1: 89.41% M2: 10.59%
M1: 96.15% M2: 3.85%
M1: 86.47% M2: 13.53%
M1: 97.18% M2: 2.82%
M1: 90.73% M2: 9.27%
M1: 96.37% M2: 3.63%
M1: 92.35% M2: 7.65%
U-2 OS
M1: 95.09% M2: 4.91%
M1: 84.09% M2: 15.91%
M1: 95.49% M2: 4.51%
M1: 85.62% M2: 14.38%
M1: 95.25% M2: 4.75%
M1: 93.30% M2: 6.70%
M1: 94.39% M2: 5.61%
M1: 93.41% M2: 6.59%
U-251 MG
M1: 96.71% M2: 3.29%
M1: 81.80% M2: 18.20%
M1: 93.68% M2: 6.32%
M1: 80.12% M2: 19.88%
M1: 91.25% M2: 8.75%
M1: 69.83% M2: 30.17%
M1: 93.07% M2: 6.93%
M1: 66.63% M2: 33.37%
Count
ROS
Neg.
Pos.
ROS(-)
ROS(+)
###
[%]
CTR DOX AZA MIX CTR DOX AZA MIX
C-NIC D-NIC

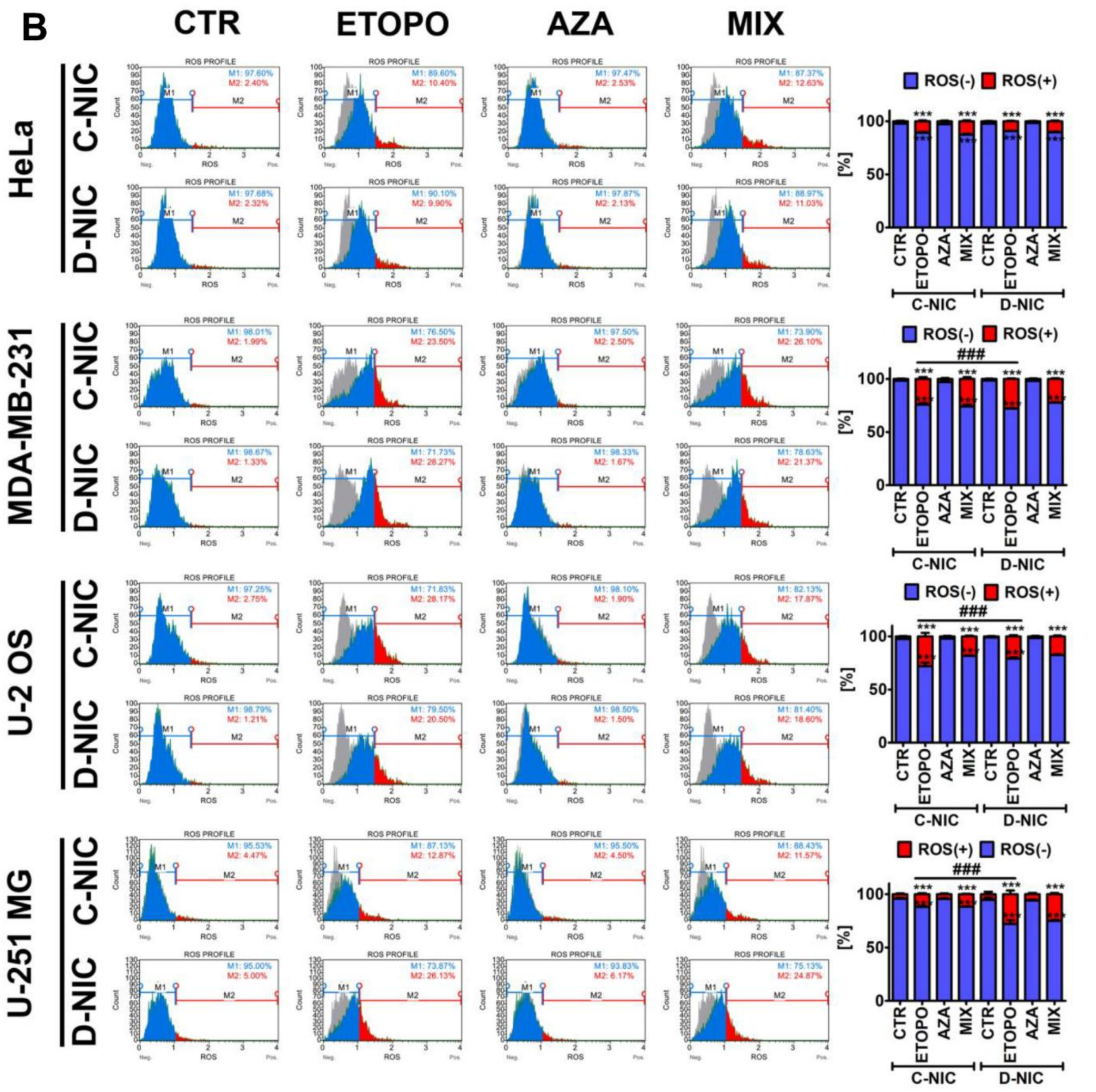


**Supplementary Figure 4.** *DNMT2/TRDMT1* gene knockout-mediated oxidative stress in four cancer cell lines, namely HeLa cervical cancer cells, MDA-MB-231 breast cancer cells, U-2 OS osteosarcoma cells and U-251 MG glioblastoma cells treated with DOX (**A**) or ETOPO (**B**) for 24 h. Superoxide levels were evaluated using flow cytometry. Representative histograms are shown. A gray control histogram is overlayed on each sample. M1, superoxide-negative subpopulation (blue); M2, superoxide-positive subpopulation (red). Bars indicate SD, n = 3, $^{***}p < 0.001$, $^{**}p < 0.01$ compared to CTR (ANOVA and Dunnett's *a posteriori* test), $^{###}p < 0.001$ compared to drug-treated C-NIC cells (ANOVA and Tukey's *a posteriori* test). CTR, control conditions; DOX, doxorubicin treatment; ETOPO, etoposide treatment; AZA, azacytidine treatment; MIX, azacytidine post-treatment; C-NIC, control cells with unmodified levels of DNMT2/TRDMT1 containing control plasmid; D-NIC, cells with *DNMT2/TRDMT1* gene knockout containing dedicated DNMT2 double nickase plasmid.

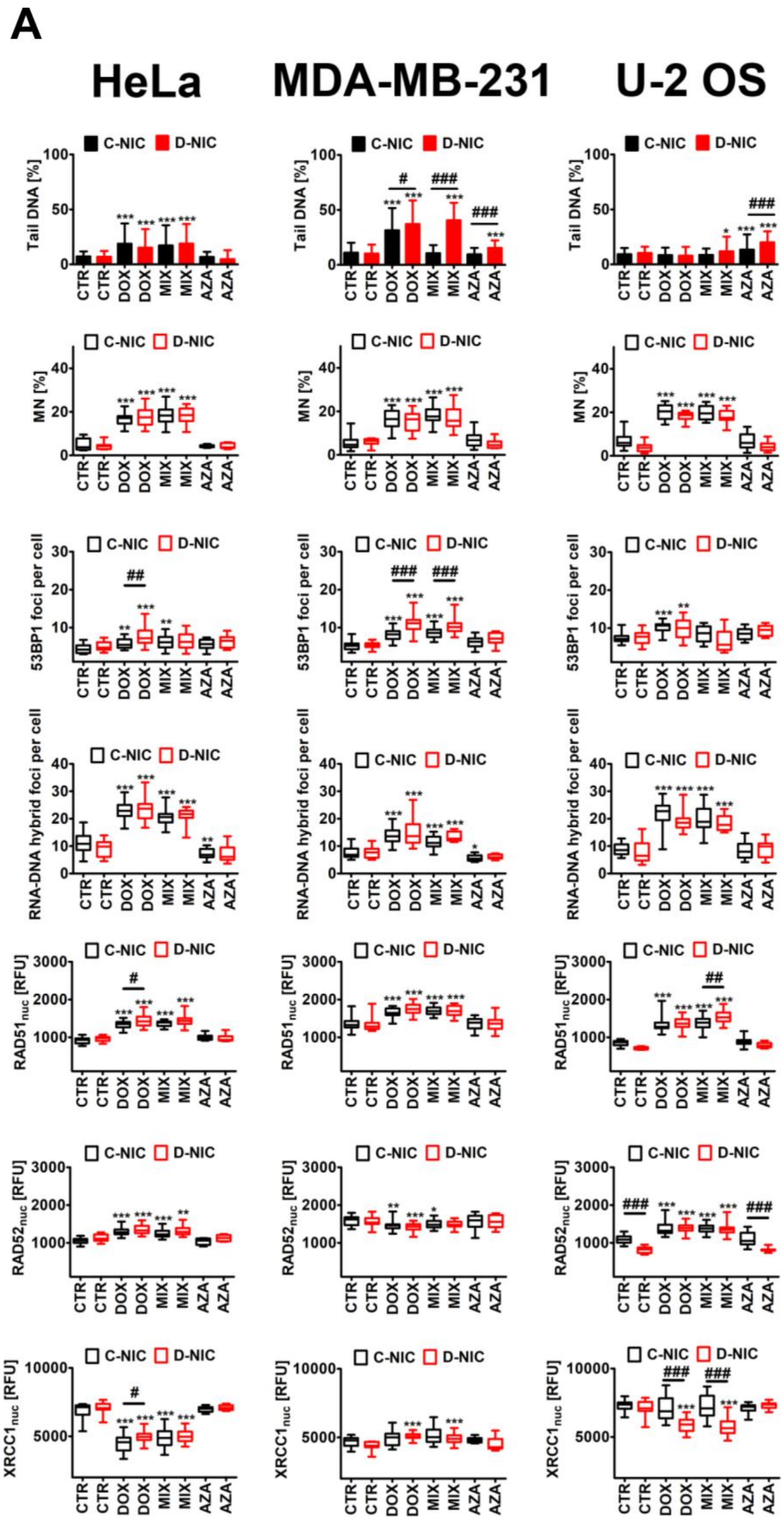
A
HeLa
MDA-MB-231
U-2 OS
C-NIC
D-NIC
Tail DNA [%]
MN [%]
53BP1 foci per cell
RNA-DNA hybrid foci per cell
$RAD51_{nuc}$ [RFU]
$RAD52_{nuc}$ [RFU]
$XRCC1_{nuc}$ [RFU]
CTR
DOX
MIX
AZA

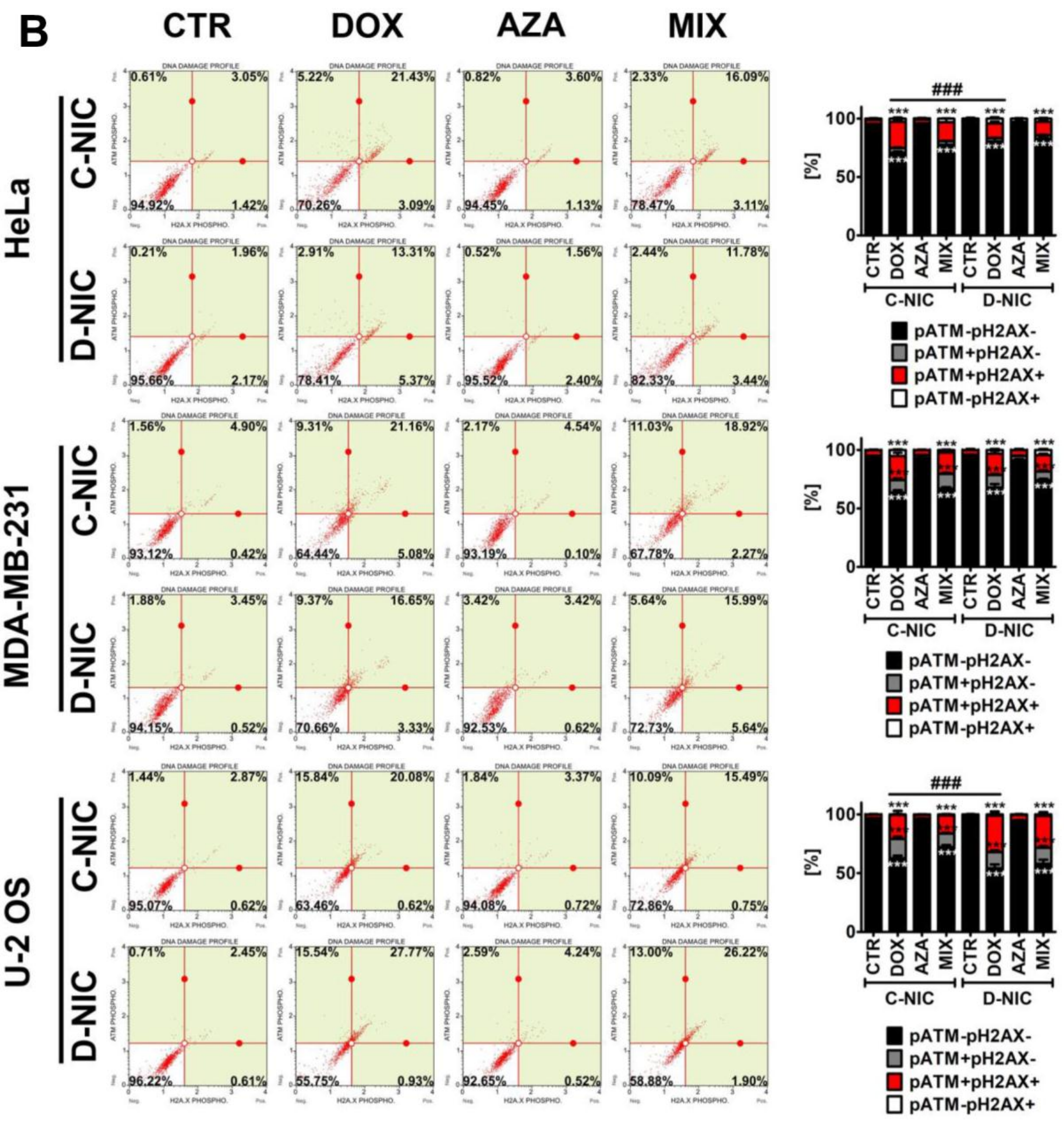
B
CTR
DOX
AZA
MIX
HeLa
MDA-MB-231
U-2 OS
C-NIC
D-NIC
DNA DAMAGE PROFILE
ATM PHOSPHO.
H2A.X PHOSPHO.
0.61% 3.05% 94.92% 1.42%
5.22% 21.43% 70.26% 3.09%
0.82% 3.60% 94.45% 1.13%
2.33% 16.09% 78.47% 3.11%
0.21% 1.96% 95.66% 2.17%
2.91% 13.31% 78.41% 5.37%
0.52% 1.56% 95.52% 2.40%
2.44% 11.78% 82.33% 3.44%
1.56% 4.90% 93.12% 0.42%
9.31% 21.16% 64.44% 5.08%
2.17% 4.54% 93.19% 0.10%
11.03% 18.92% 67.78% 2.27%
1.88% 3.45% 94.15% 0.52%
9.37% 16.65% 70.66% 3.33%
3.42% 3.42% 92.53% 0.62%
5.64% 15.99% 72.73% 5.64%
1.44% 2.87% 95.07% 0.62%
15.84% 20.08% 63.46% 0.62%
1.84% 3.37% 94.08% 0.72%
10.09% 15.49% 72.86% 0.75%
0.71% 2.45% 96.22% 0.61%
15.54% 27.77% 55.75% 0.93%
2.59% 4.24% 92.65% 0.52%
13.00% 26.22% 58.88% 1.90%
[%]
###
***
CTR DOX AZA MIX CTR DOX AZA MIX
C-NIC D-NIC
pATM-pH2AX-
pATM+pH2AX-
pATM+pH2AX+
pATM-pH2AX+

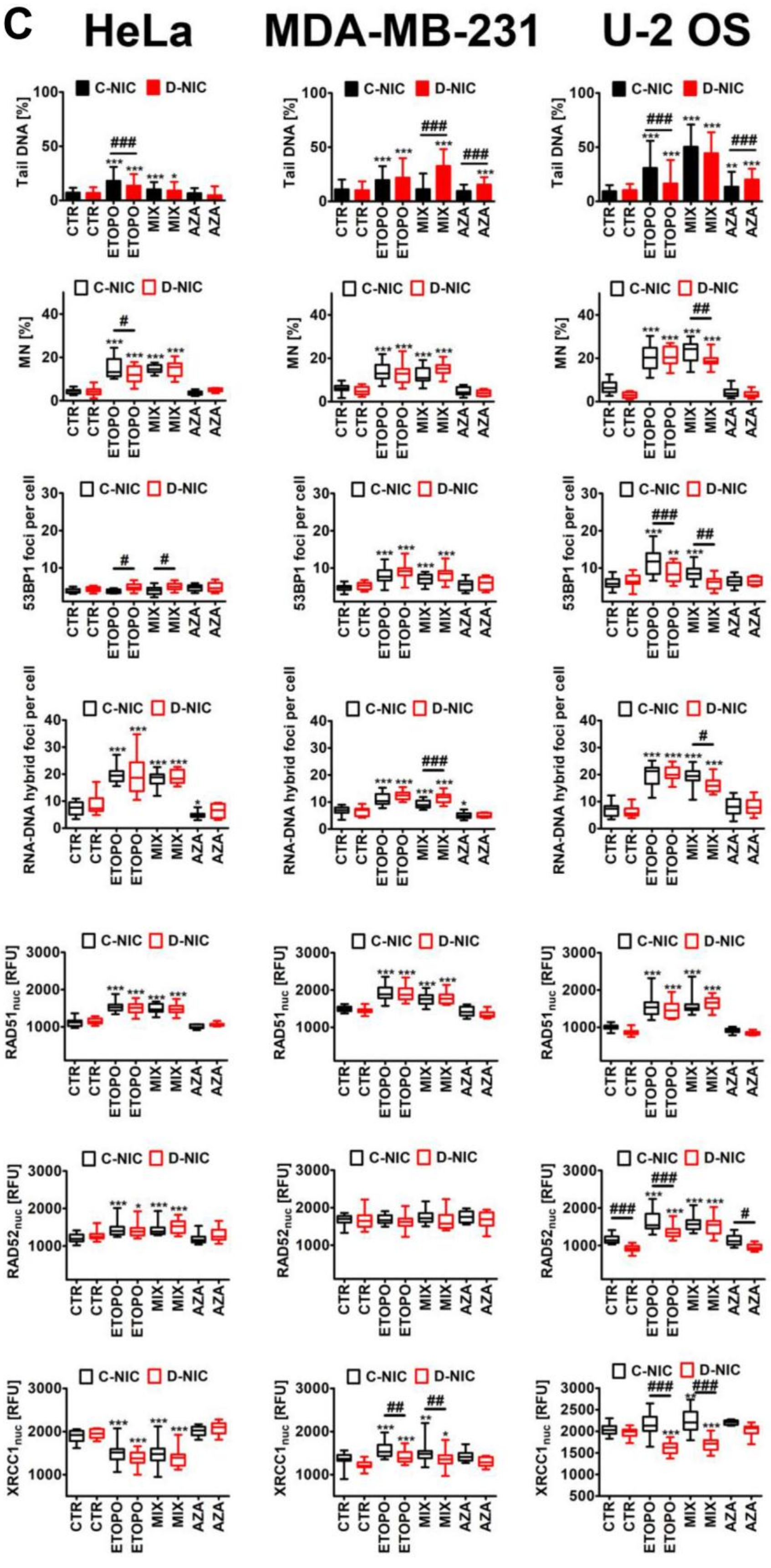
C
HeLa
MDA-MB-231
U-2 OS
C-NIC
D-NIC
Tail DNA [%]
MN [%]
53BP1 foci per cell
RNA-DNA hybrid foci per cell
RAD51nuc [RFU]
RAD52nuc [RFU]
XRCC1nuc [RFU]
CTR
ETOPO
MIX
AZA

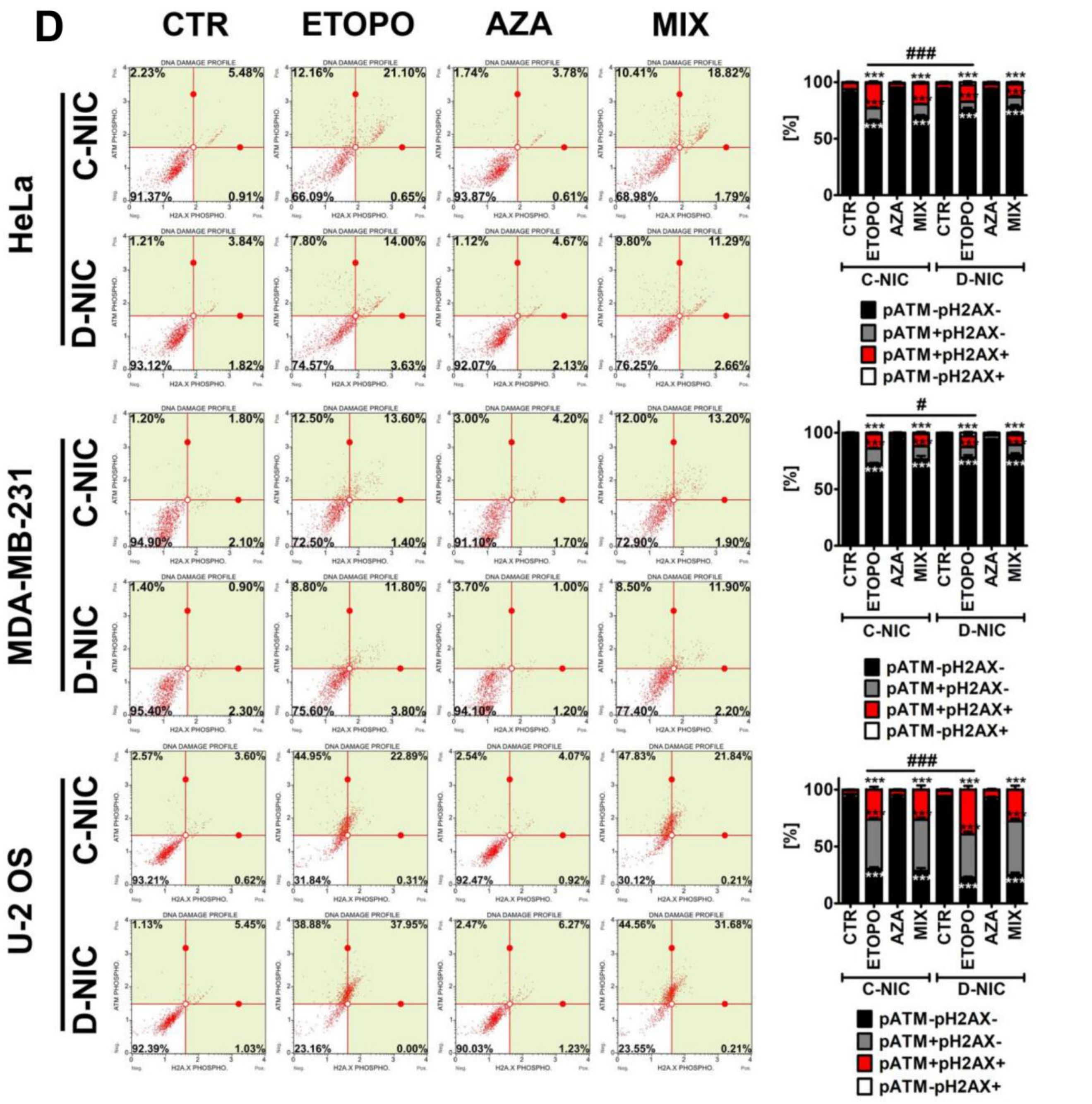


**Supplementary Figure 5.** *DNMT2/TRDMT1* gene knockout-mediated DNA damage, chromosomal damage and DNA damage response (DDR) in three cancer cell lines, namely HeLa cervical cancer cells, MDA-MB-231 breast cancer cells and U-2 OS osteosarcoma cells treated with DOX (**A**, **B**) or ETOPO (**C**, **D**). (**A**, **C**) DNA double-strands breaks (DSBs) as tail DNA (%) were assessed using neutral comet assay. Bars indicate SD, n = 3, $^{***}p < 0.001$, $^{**}p < 0.01$ $^{*}p < 0.05$ compared to CTR (ANOVA and Dunnett's *a posteriori* test), $^{###}p < 0.001$, $^{##}p < 0.01$ compared to C-NIC cells at the same culture conditions (ANOVA and Tukey's *a posteriori* test). Micronuclei (MN) formation was assayed using Hoechst 33342 staining and scored as %. 53BP1 foci, RNA-DNA hybrid foci, RAD51, RAD52 and XRCC1 immunostaining. The levels of RAD51, RAD52 and XRCC1 are expressed as relative fluorescence units (RFU). Box and whisker plots are shown, n = 3, $^{***}p < 0.001$, $^{**}p < 0.01$, $^{*}p <$ 0.05 compared to CTR (ANOVA and Dunnett's *a posteriori* test), $^{###}p < 0.001$, $^{##}p < 0.01$, $^{#}p < 0.05$ compared to C-NIC cells at the same culture conditions (ANOVA and Tukey's *a posteriori* test). (**B**, **D**) Activation of ATM and H2AX was evaluated using flow cytometry. Representative dot plots are shown. Bars indicate SD, n = 3, $^{***}p < 0.001$ compared to CTR (ANOVA and Dunnett's *a posteriori* test), $^{###}p < 0.001$, $^{#}p < 0.05$ compared to drug-treated C-NIC cells (ANOVA and Tukey's *a posteriori* test). CTR, control conditions; DOX, doxorubicin treatment; ETOPO, etoposide treatment; AZA, azacytidine treatment; MIX, azacytidine post-treatment; C-NIC, control cells with unmodified levels of DNMT2/TRDMT1 containing control plasmid; D-NIC, cells with *DNMT2/TRDMT1* gene knockout containing dedicated DNMT2 double nickase plasmid.

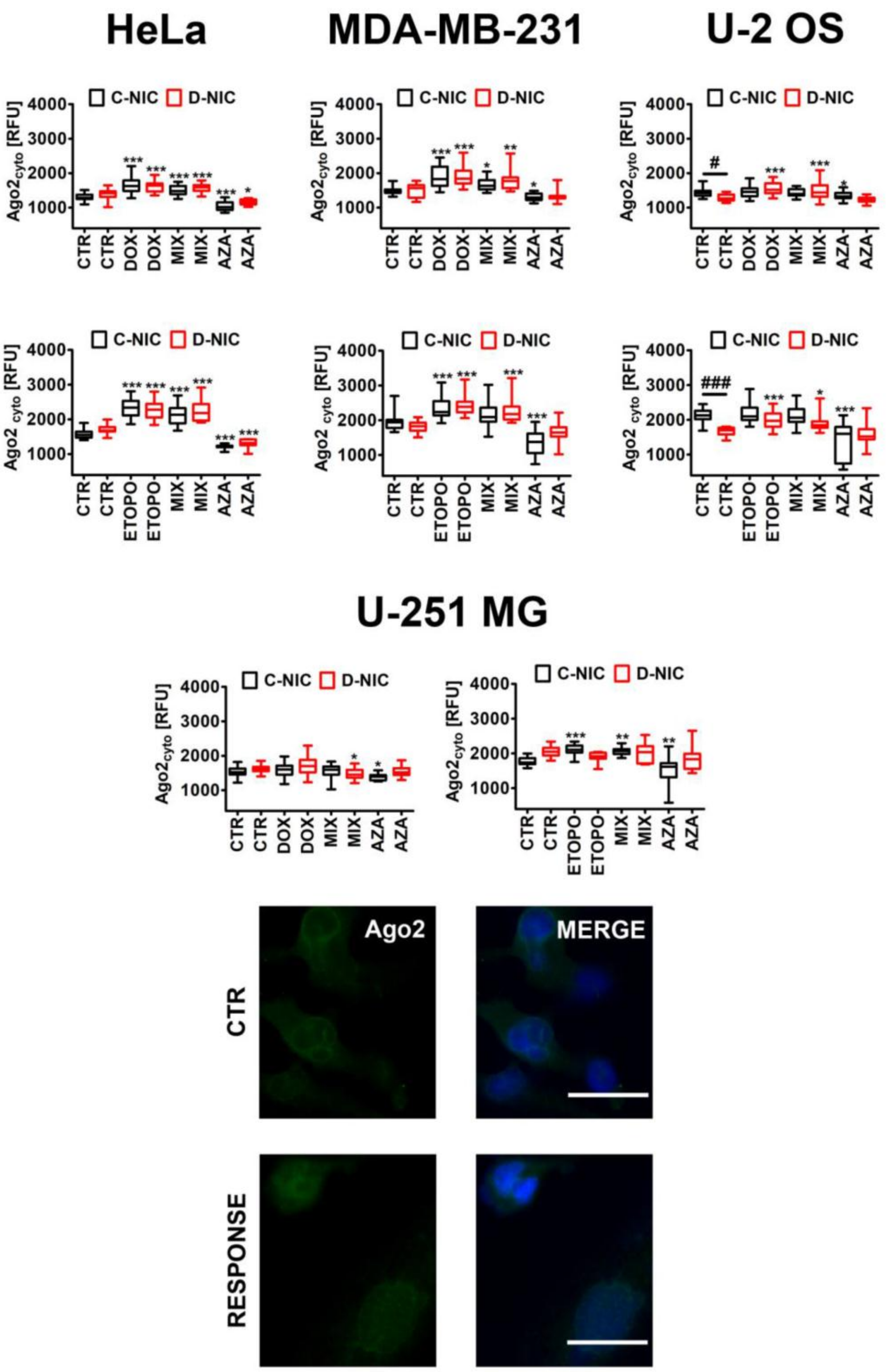


**Supplementary Figure 6. *DNMT2/TRDMT1* gene knockout-mediated changes in the levels of Ago2 in four cancer cell lines, namely HeLa cervical cancer cells, MDA-MB-231 breast cancer cells, U-2 OS osteosarcoma cells and U-251 MG glioblastoma cells treated with DOX or ETOPO.** The levels of Ago2 are expressed as relative fluorescence units (RFU). Box and whisker plots are shown, n = 3, $^{***}p < 0.001$, $^{**}p < 0.01$, $^{*}p < 0.05$ compared to CTR (ANOVA and Dunnett's *a posteriori* test), $^{\#\#\#}p < 0.001$, $^{\#}p < 0.05$ compared to C-NIC cells at the same culture conditions (ANOVA and Tukey's *a posteriori* test). Ago2 immunostaining (green). Representative microphotographs are shown, objective 20x, nucleus staining (blue), RESPONSE, representative DOX or ETOPO treatment. CTR, control conditions; DOX, doxorubicin treatment; ETOPO, etoposide treatment; AZA, azacytidine treatment; MIX, azacytidine post-treatment; C-NIC, control cells with unmodified levels of DNMT2/TRDMT1 containing control plasmid; D-NIC, cells with *DNMT2/TRDMT1* gene knockout containing dedicated DNMT2 double nickase plasmid.

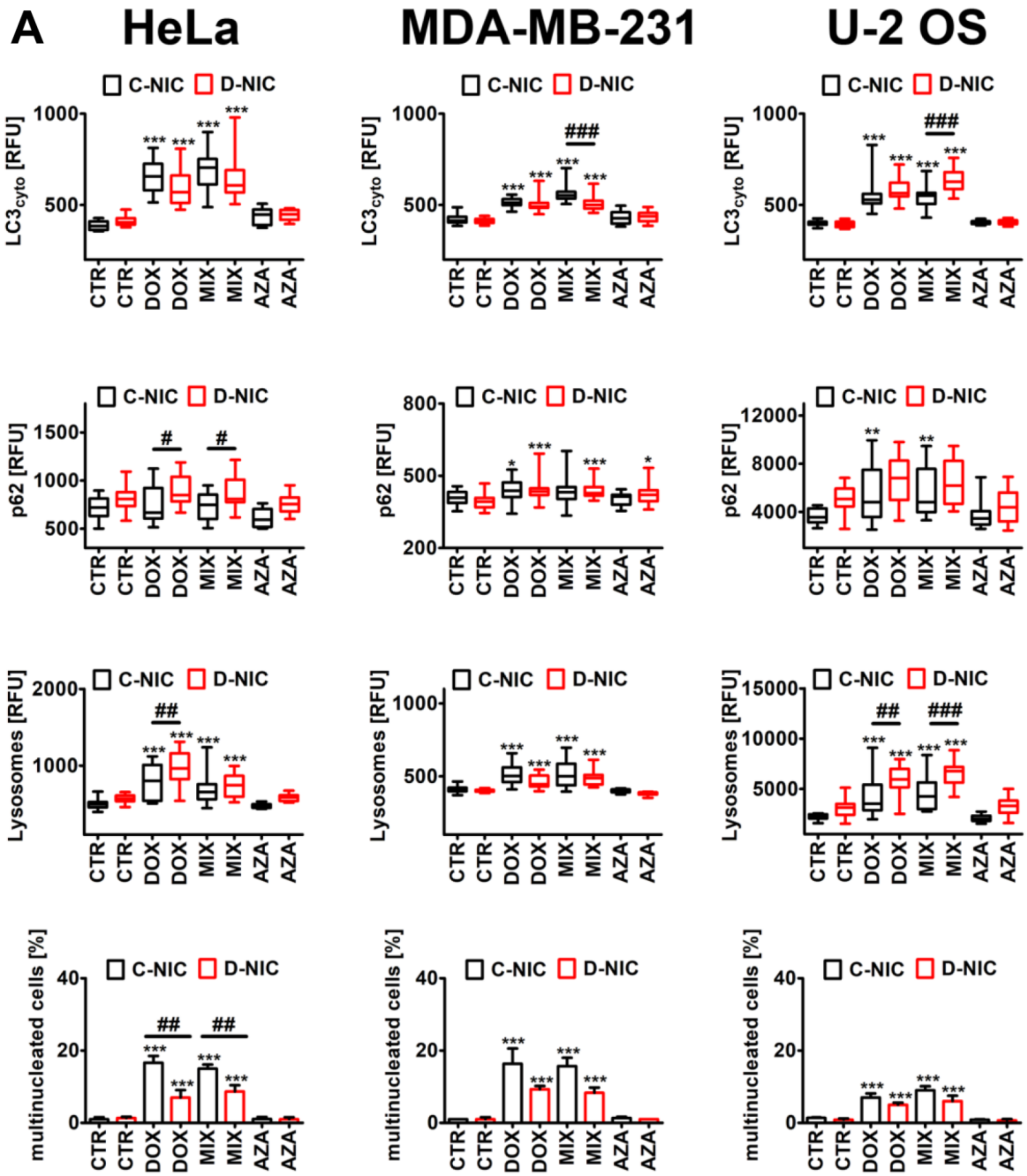
A
HeLa
MDA-MB-231
U-2 OS
C-NIC
D-NIC
LC3cyto [RFU]
p62 [RFU]
Lysosomes [RFU]
multinucleated cells [%]
CTR
DOX
MIX
AZA

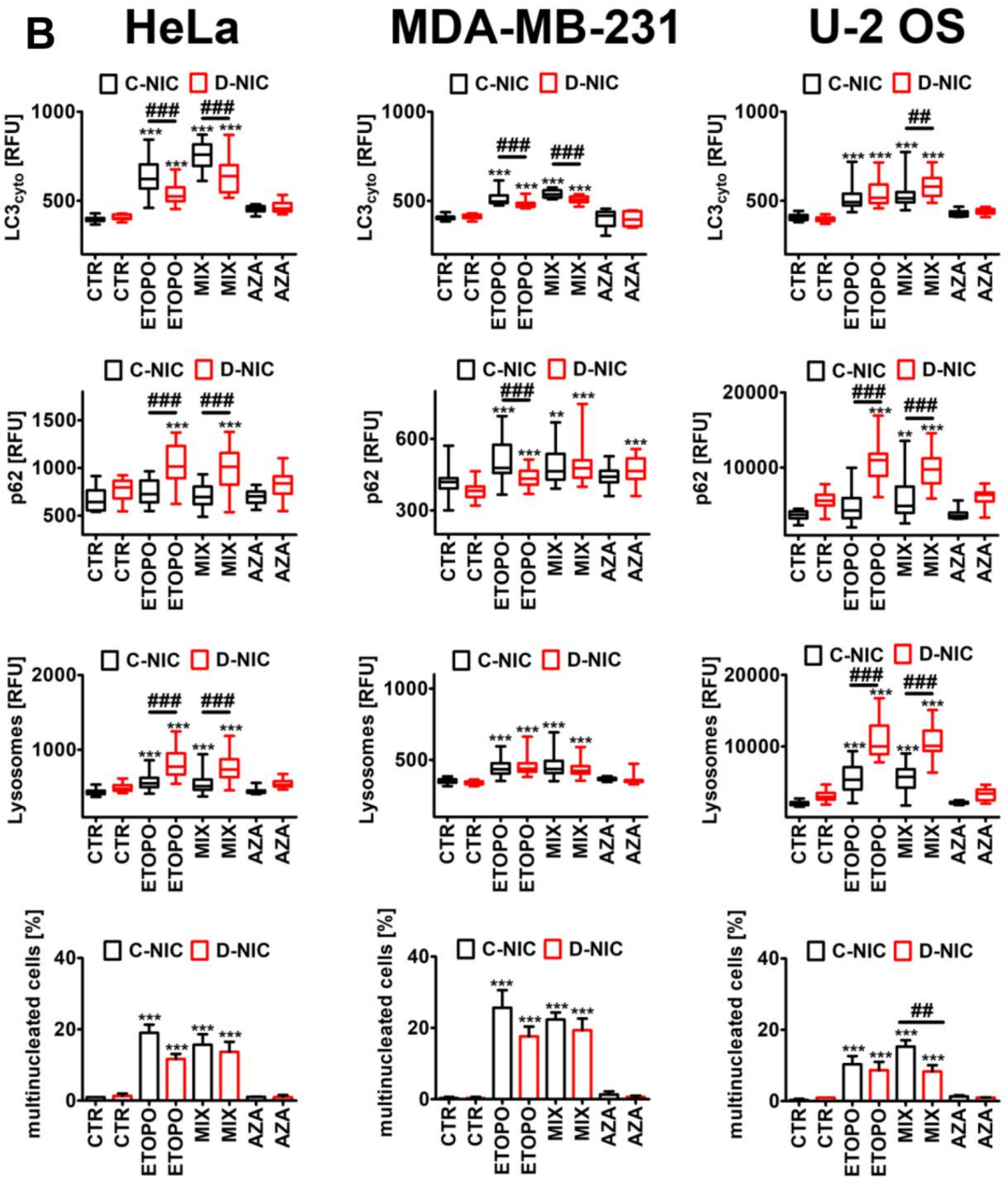


**Supplementary Figure 7.** *DNMT2/TRDMT1* gene knockout-mediated autophagy and multinucleation in three cancer cell lines, namely HeLa cervical cancer cells, MDA-MB-231 breast cancer cells and U-2 OS osteosarcoma cells treated with DOX (**A**) or ETOPO (**B**). LC3 immunostaining and GFP-based imaging of a lysosomal marker Lamp1 and an autophagy marker p62. The levels of LC3, p62 and Lamp1 are expressed as relative fluorescence units (RFU). Box and whisker plots are shown, n = 3, $^{***}p < 0.001$, $^{**}p < 0.01$, $^{*}p < 0.05$ compared to CTR (ANOVA and Dunnett's *a posteriori* test), $^{\#\#\#}p < 0.001$, $^{\#\#}p < 0.01$, $^{\#}p < 0.05$ compared to C-NIC cells at the same culture conditions (ANOVA and Tukey's *a posteriori* test). Multinucleation events (%) were analyzed using Hoechst 33342 staining. Bars indicate SD, n = 3, $^{***}p < 0.001$ compared to CTR (ANOVA and Dunnett's *a posteriori* test), $^{\#\#}p < 0.01$ compared to C-NIC cells at the same culture conditions (ANOVA and Tukey's *a posteriori* test). CTR, control conditions; DOX, doxorubicin treatment; ETOPO, etoposide treatment; AZA, azacytidine treatment; MIX, azacytidine post-treatment; C-NIC, control cells with unmodified levels of DNMT2/TRDMT1 containing control plasmid; D-NIC, cells with *DNMT2/TRDMT1* gene knockout containing dedicated DNMT2 double nickase plasmid.

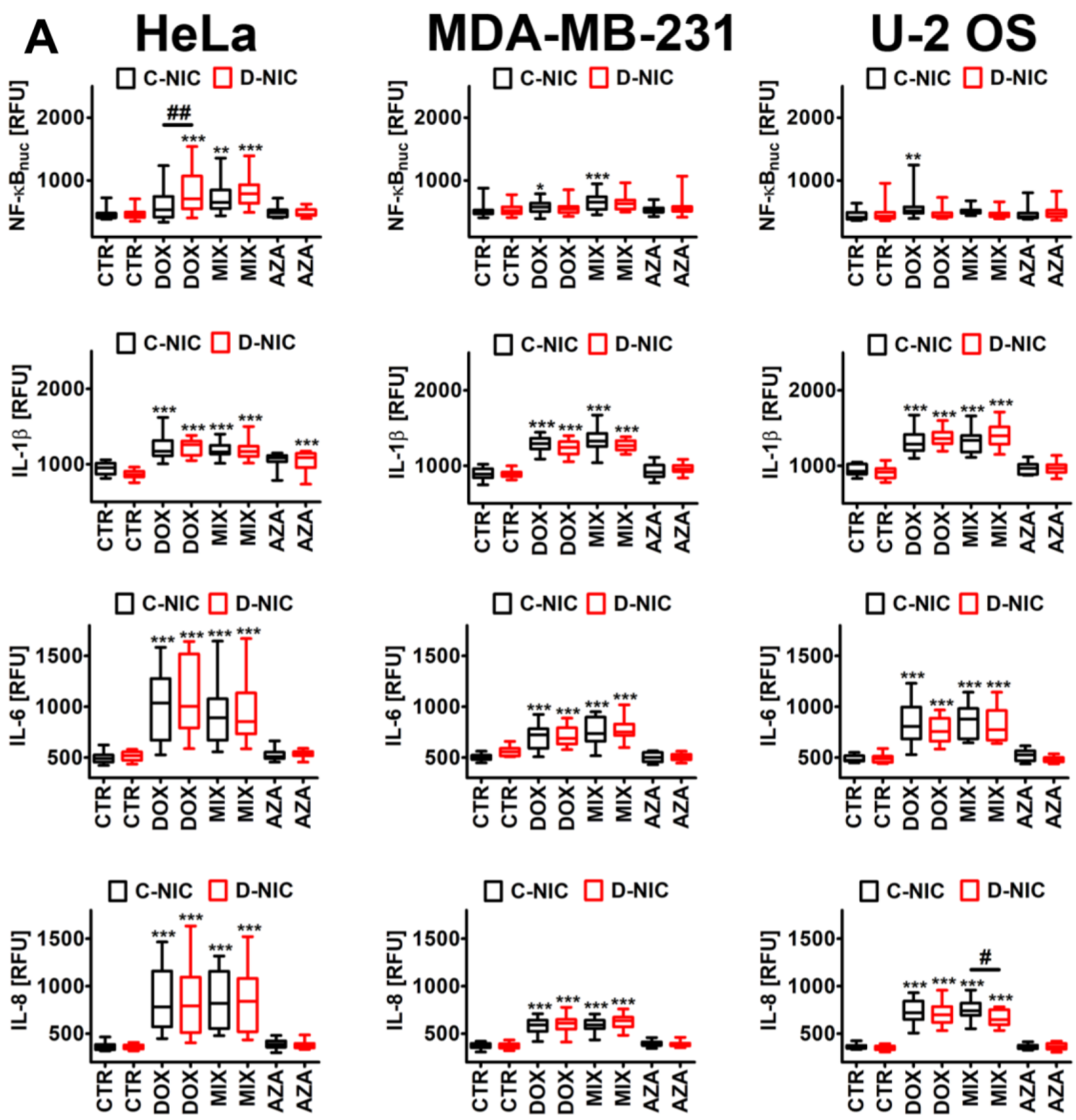
A
HeLa
MDA-MB-231
U-2 OS
C-NIC
D-NIC
NF-κB$_{nuc}$ [RFU]
IL-1β [RFU]
IL-6 [RFU]
IL-8 [RFU]
CTR
DOX
MIX
AZA
##
#

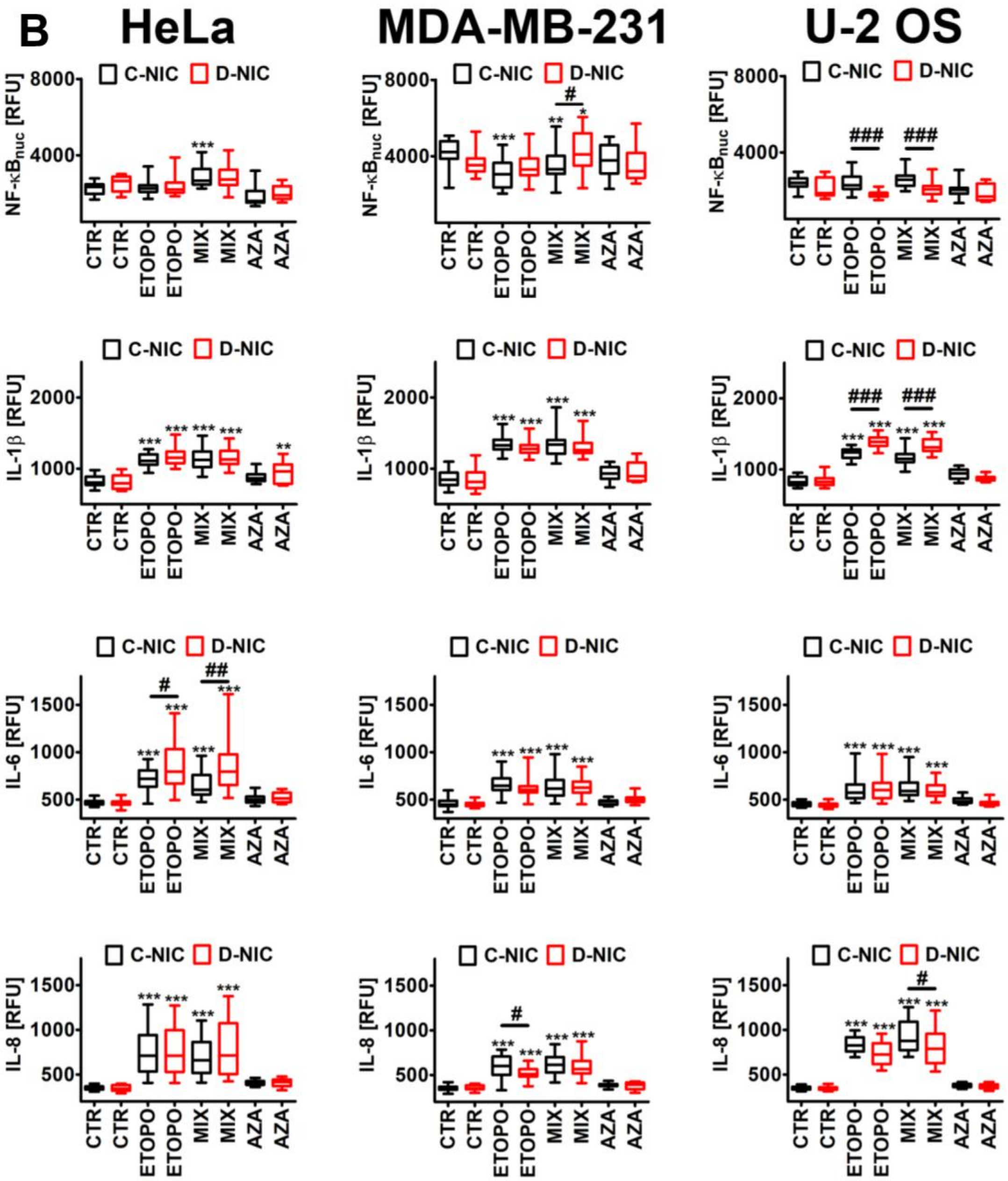


**Supplementary Figure 8.** *DNMT2/TRDMT1* gene knockout-mediated senescence-associated secretory phenotype (SASP) in three cancer cell lines, namely HeLa cervical cancer cells, MDA-MB-231 breast cancer cells and U-2 OS osteosarcoma cells treated with DOX (**A**) or ETOPO (**B**). The levels of NF-κB, IL-1β, IL-6 and IL-8 are expressed as relative fluorescence units (RFU). Box and whisker plots are shown, n = 3, $^{***}p < 0.001$, $^{**}p < 0.01$, $^{*}p < 0.05$ compared to CTR (ANOVA and Dunnett's *a posteriori* test), $^{\#\#\#}p < 0.001$, $^{\#\#}p < 0.01$, $^{\#}p < 0.05$ compared to C-NIC cells at the same culture conditions (ANOVA and Tukey's *a posteriori* test). CTR, control conditions; DOX, doxorubicin treatment; ETOPO, etoposide treatment; AZA, azacytidine treatment; MIX, azacytidine post-treatment; C-NIC, control cells with unmodified levels of DNMT2/TRDMT1 containing control plasmid; D-NIC, cells with *DNMT2/TRDMT1* gene knockout containing dedicated DNMT2 double nickase plasmid.

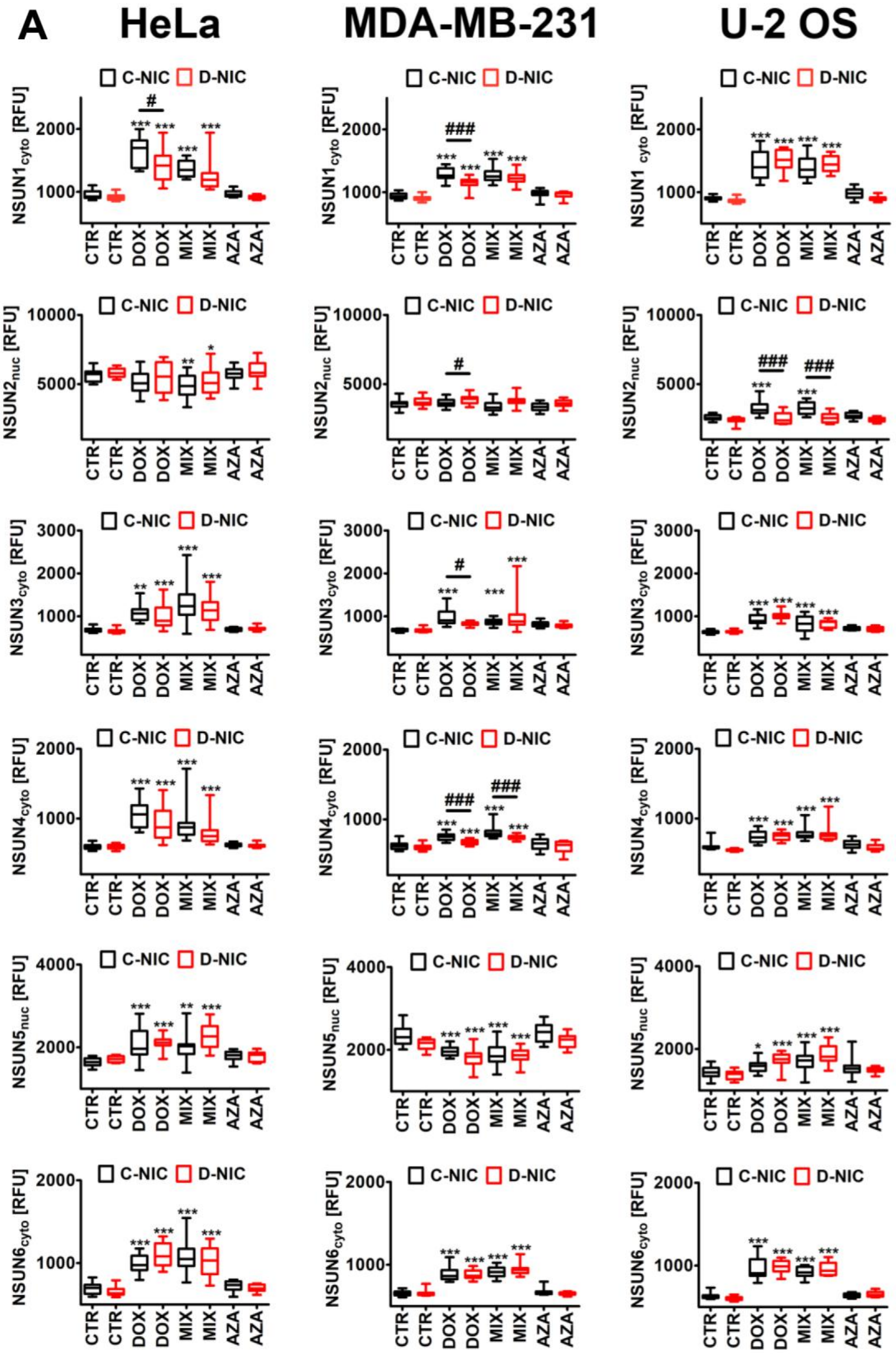
A
HeLa
MDA-MB-231
U-2 OS
C-NIC
D-NIC
NSUN1cyto [RFU]
NSUN2nuc [RFU]
NSUN3cyto [RFU]
NSUN4cyto [RFU]
NSUN5nuc [RFU]
NSUN6cyto [RFU]
CTR
DOX
MIX
AZA

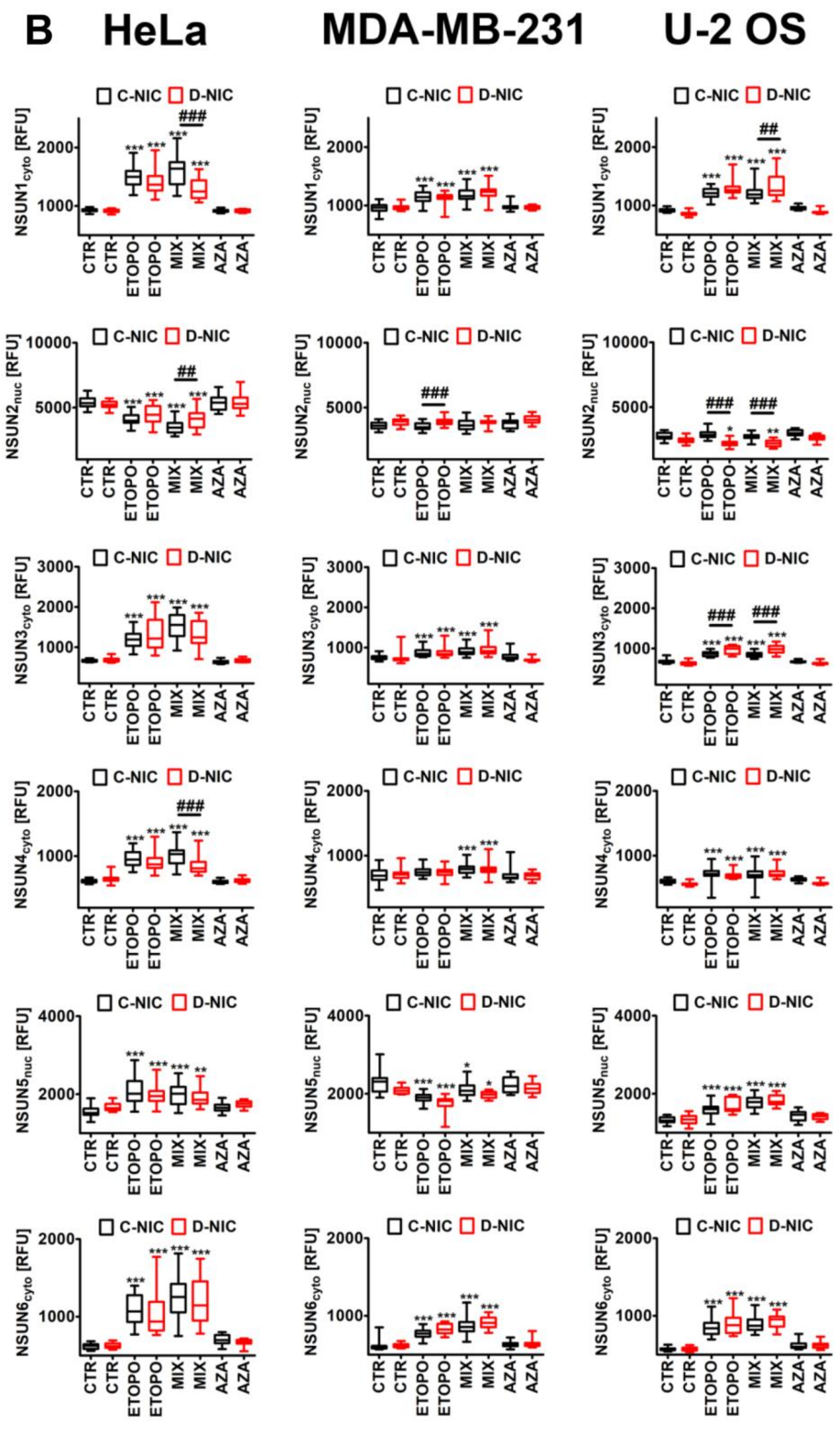


**Supplementary Figure 9.** *DNMT2/TRDMT1* gene knockout-mediated changes in the levels of NSUN proteins in three cancer cell lines, namely HeLa cervical cancer cells, MDA-MB-231 breast cancer cells and U-2 OS osteosarcoma cells treated with DOX (**A**) or ETOPO (**B**). The levels of NSUN1, NSUN2, NSUN3, NSUN4, NSUN5 and NSUN6 are expressed as relative fluorescence units (RFU). Box and whisker plots are shown, n = 3, $^{***}p < 0.001$, $^{**}p < 0.01$, $^{*}p < 0.05$ compared to CTR (ANOVA and Dunnett's *a posteriori* test), $^{\#\#\#}p < 0.001$, $^{\#\#}p < 0.01$, $^{\#}p < 0.05$ compared to C-NIC cells at the same culture conditions (ANOVA and Tukey's *a posteriori* test). CTR, control conditions; DOX, doxorubicin treatment; ETOPO, etoposide treatment; AZA, azacytidine treatment; MIX, azacytidine post-treatment; C-NIC, control cells with unmodified levels of DNMT2/TRDMT1 containing control plasmid; D-NIC, cells with *DNMT2/TRDMT1* gene knockout containing dedicated DNMT2 double nickase plasmid.

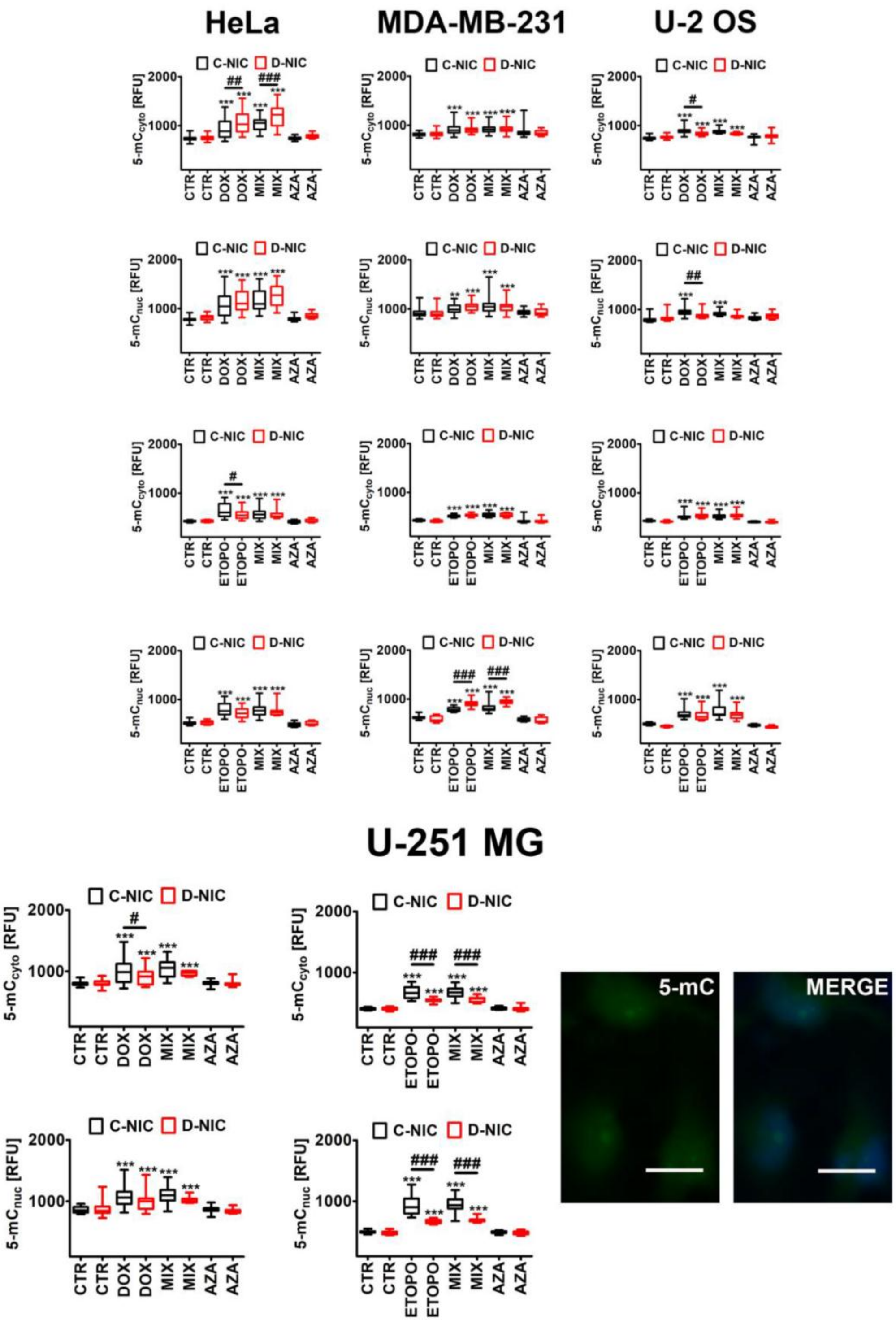


**Supplementary Figure 10. *DNMT2/TRDMT1* gene knockout-mediated changes in the levels of cytosolic and nuclear fractions of 5-methylcytosine (5-mC) in four cancer cell lines, namely HeLa cervical cancer cells, MDA-MB-231 breast cancer cells, U-2 OS osteosarcoma cells and U-251 MG glioblastoma cells treated with DOX or ETOPO.** The levels of cytosolic and nuclear 5-mC are expressed as relative fluorescence units (RFU). Box and whisker plots are shown, n = 3, $^{***}p < 0.001$, $^{**}p < 0.01$ compared to CTR (ANOVA and Dunnett's *a posteriori* test), $^{\#\#\#}p < 0.001$, $^{\#\#}p < 0.01$, $^{\#}p < 0.05$ compared to C-NIC cells at the same culture conditions (ANOVA and Tukey's *a posteriori* test). 5-mC immunostaining (green). Representative microphotographs are shown, objective 20x, nucleus staining (blue). CTR, control conditions; DOX, doxorubicin treatment; ETOPO, etoposide treatment; AZA, azacytidine treatment; MIX, azacytidine post-treatment; C-NIC, control cells with unmodified levels of DNMT2/TRDMT1 containing control plasmid; D-NIC, cells with *DNMT2/TRDMT1* gene knockout containing dedicated DNMT2 double nickase plasmid.
